# Self-reported symptoms and health complaints associated with exposure to *Ixodes ricinus*-borne pathogens

**DOI:** 10.1186/s13071-022-05228-4

**Published:** 2022-03-18

**Authors:** Tal Azagi, Margriet Harms, Arno Swart, Manoj Fonville, Dieuwertje Hoornstra, Lapo Mughini-Gras, Joppe W. Hovius, Hein Sprong, Cees van den Wijngaard

**Affiliations:** 1 Centre for Infectious Diseases Research, National Institute for Public Health and the Environment, P.O. Box 1, Bilthoven, 3720 BA The Netherlands; 2 Center for Experimental and Molecular Medicine, Amsterdam University Medical Centers Location, Academic Medical Center, Meibergdreef 9, 1105 AZ Amsterdam, The Netherlands

**Keywords:** Tick-borne diseases, *Ixodes ricinus*

## Abstract

**Background:**

The impact of infections with tick-borne pathogens (TBPs) other than *Borrelia burgdorferi* (s.l.) and tick-borne encephalitis virus (TBEV) on public health in Europe remains unclear. Our goal is to evaluate whether the presence of these TBPs in ticks can be associated with self-reported health complaints.

**Methods:**

We enrolled individuals who were bitten by *I. ricinus* between 2012 and 2015 and collected their relevant demographic and clinical information using a self-administered online questionnaire. A total of 4163 *I. ricinus* ticks sent by the participants were subject to molecular analyses for detection of specific TBPs. Associations between the presence of TBPs in ticks and self-reported complaints and symptoms were evaluated by means of a stepwise approach using a generalized linear model (GLM).

**Results:**

Of 17 self-reported complaints and symptoms significant in the univariate analyses, 3 had a highly significant association (*P* < 0.01) with at least one TBP in the multivariate analysis. Self-reported Lyme borreliosis was significantly associated (*P* < 0.001) with *B. burgdorferi* (s.l.) infection. Facial paralysis was associated (*P* < 0.01) with infection with *B. miyamotoi, N. mikurensis* and *R. helvetica*. Finally, a significant association (*P* < 0.001) was found between nocturnal sweating and *A. phagocytophilum*.

**Conclusions:**

We found associations between the presence of TBPs in ticks feeding on humans and self-reported symptoms. Due to the subjective nature of such reports and the fact that infection was determined in the ticks and not in the patient samples, further prospective studies utilizing diagnostic modalities should be performed before any clinical outcome can be causally linked to infection with TBPs.

**Graphical Abstract:**

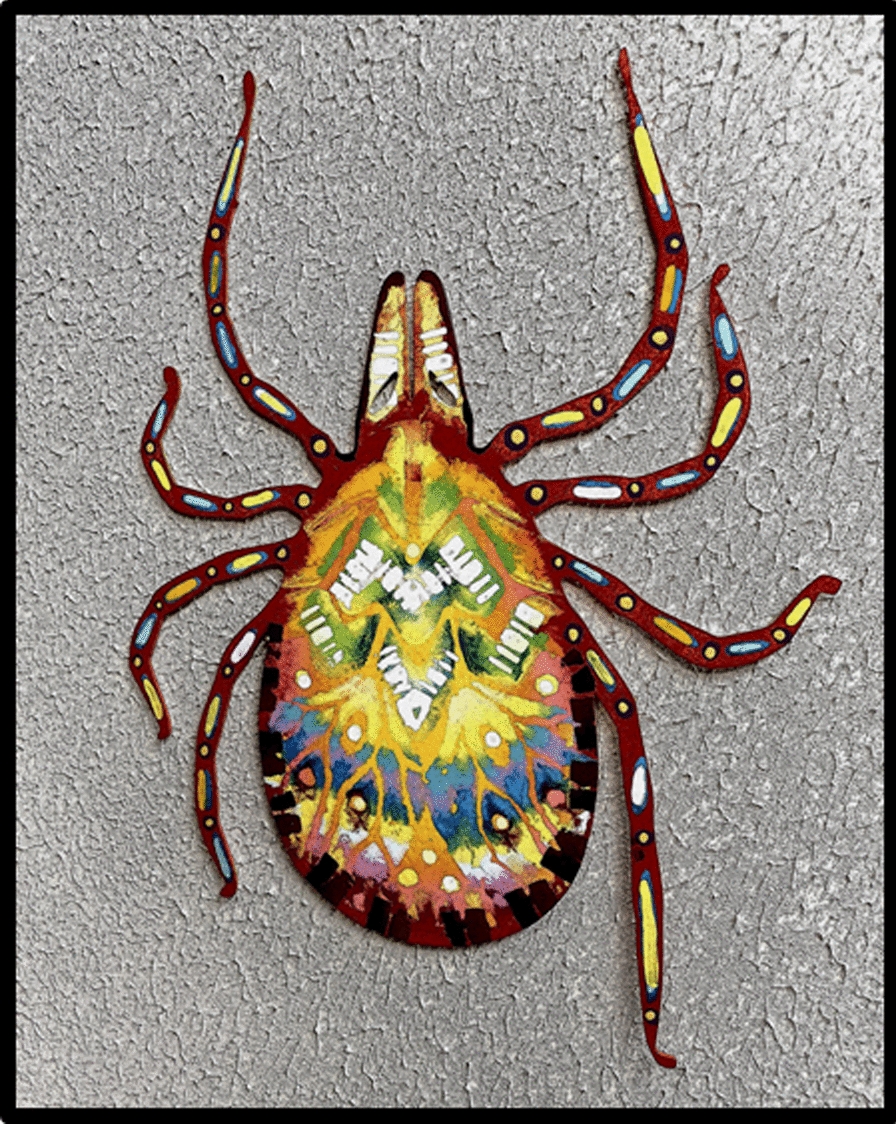

**Supplementary Information:**

The online version contains supplementary material available at 10.1186/s13071-022-05228-4.

## Introduction

Despite numerous publications describing the presence of potentially pathogenic microorganisms in *Ixodes ricinus* and a trend of tick population growth and geographic expansion [1], the impact of infections with tick-borne pathogens other than *Borrelia* *burgdorferi* sensu lato (s.l.) and tick-borne encephalitis virus (TBEV) on public health remains to be elucidated in Europe [2, 3].

Studies have shown that there is substantial exposure of humans to *I. ricinus*-borne microorganisms through tick bites, such as *Borrelia miyamotoi*, *Anaplasma phagocytophilum, Babesia* (s.s.), *Babesia microti*, *Neoehrlichia mikurensis*, *Rickettsia helvetica* and *Spiroplasma ixodetis* [2, 4]. However, for some of these tick-borne pathogens (TBPs), the pathogenicity remains questionable and only few well described cases exist [5]. In The Netherlands, the risk of human infection with at least one of these TBPs from a tick bite has been estimated at roughly 2.4% based on molecular detection in blood samples collected after the tick bite [2, 6]. Nevertheless, despite an increase in reported tick bites in the general population [7] over the last decades, only two autochthonous anaplasmosis and two *B. miyamotoi* case reports have been published in the literature so far [8–11]. A Dutch prospective study focusing on the well-established TBP *B*. *burgdorferi* (s.l.) found that the risk for either Lyme borreliosis (LB) or seroconversion after a tick bite was 5.1%, while the risk of LB after a tick bite was 2.6% [12]. If this would be representative for other TBPs, it could be surmised that the risk of developing clinically overt disease after a tick bite with other TBPs is even lower than 2.4%.

Underdiagnosis of tick-borne illness has been linked to the presentation of self-limiting and uncharacteristic symptoms, making them hard for medical doctors to recognize or leading individuals not to seek medical help at all [2]. Furthermore, a lack of well-defined diagnostic criteria and the absence of standardized diagnostic tools in routine practice [5] mean, for the most, only certain disease cases will be thoroughly investigated using available diagnostic tests, which are only available in specialized settings [13].

The potential clinical burden of these TBPs could be measured by assessing self-reported health complaints after a tick bite, i.e., illness [14], as opposed to looking at more defined and medically diagnosed cases, i.e., disease [14]. This approach may enable us to deduce whether infection with TBPs other than *B*. *burgdorferi* (s.l.) and TBEV could be associated with certain health complaints after a tick bite, which could aid in providing an estimation of the extent of underdiagnosed mild tick-borne related illness in the population.

In an attempt to provide insights into the clinical burden of these TBPs, we used a citizen science approach to enroll individuals who were bitten by *I. ricinus* and collected their relevant demographic and clinical information using a self-administered questionnaire, while the detached ticks were subjected to molecular analyses for detection of TBPs.

## Materials and methods

### Study design and questionnaires

Between the years 2012 and 2015, participants reported tick bites through the website www.tekenradar.nl and sent the detached ticks to the Dutch National Institute of Public Health and the Environment (RIVM) for analysis. After 3 months from enrollment, the participants were asked to fill in a questionnaire (Additional file 2) regarding health complaints experienced since the reported tick bite. Participants were made aware of the results only if the tick was positive for *B. burgdorferi* (s.l.) and not earlier than 9 months after the completion of the first questionnaire.

### Data

#### Survey data

For each participant, presence or absence of 33 self-reported complaints and symptoms at 3 months after the tick bite event was obtained from the completed questionnaire (Additional file 1). Participants who reported a second tick bite within the 3 months of the first notification were excluded from the analysis. Out of 7317 initial inclusions, 4163 participants who completed the 3-month questionnaire were included in this study. Reported LB and erythema migrans (EM) were highly correlated and grouped as “LB/EM.” Since for this outcome *B. burgdorferi* (s.l.) is the known causative pathogen, we used this to validate whether our analysis identified the correct causative pathogen. “Fever” and “elevated body temperature” were highly correlated as well and grouped as “fever.” For some LB/EM self-reported cases a general practitioner (GP) confirmation was obtained (with patient’s consent) to confirm the diagnosis. Antibiotic use was inquired about at startup and in the questionnaires. Data on underlying health conditions were reported by the participants and translated into a scale of “frailty,” which is defined as the number of comorbidities per individual (Additional file 1). At enrollment, participants were asked for demographic data such as gender and their educational level, which was classified as low education (primary school), intermediate education (secondary school), higher education (undergraduate and graduate school) or unknown.

#### Processing of ticks and molecular detection of TBPs

Tick species, developmental stage and gender were examined by microscope and processed as previously described [15]. We included only nymph and adult *I. ricinus* ticks; all larvae (*n* = 140) were excluded because of their low number and lower infection rates. If a participant sent more than one tick per tick bite event, the ticks were pooled (*n* = 344); samples sent by participants who reported more than one tick bite event were excluded.

After arrival at the laboratory, ticks were stored at − 20 °C in 70% ethanol. DNA was extracted as previously described [15]. The lysates were stored at 4 °C. Samples were analyzed with different (multiplex or singleplex) real-time PCRs, based on various target genes for *B. burgdorferi* (s.l.), *B. miyamotoi*, *A. phagocytophilum, Babesia* (s.s.), *B. microti*, *N. mikurensis*, *Rickettsia helvetica* and *Spiroplasma ixodetis* as published before [2, 4]. Since the genes FlaB and OspA both detect the presence of *B. burgdorferi* (s.l.), their qPCR results were grouped and regarded as one pathogen.

### Statistical analysis

First, a generalized linear model (GLM) with a binary outcome and a logit link function (i.e., a logistic regression) was used to assess the odds of reporting each complaint (outcome variable) as a function of each pathogen (predictor variable). The participant’s sex and educational level were included as categorical covariates, while age and frailty were included as continuous covariates. Tick stage was included as categorical covariate as well; pools were analyzed according to the most advanced life stage. Antibiotic use was excluded from the analysis since the timing and reason for the treatment were unknown.

For each complaint, we then included all the aforementioned control covariates plus those pathogens associated with the outcome at a significance level of *P* < 0.1 at the single-variable analysis in a full GLM. Collinearity between predictor variables was checked by means of variance inflation factors (VIF), ensuring a VIF < 5 (Additional file 1). A combined backward and forward stepwise model selection procedure was then performed based on model fit (Akaike information criterion, AIC) to build a parsimonious model. Because we assessed many possible associations between complaints and pathogens, there was a relatively high chance of identifying significant associations by coincidence. Therefore, we only reported the associations with a *P*-value < 0.01, and the other associations were reported in the supplementary data. Associations were expressed as odds ratios (ORs).

All analyses were performed using R version 4.0.5 [16].

## Results

### TBPs in submitted ticks

Out of 7317 initial inclusions, a total of 4163 completed the 3-month questionnaire and sent *I. ricinus* ticks (3819 individual ticks and 344 pools), which were screened for TBPs. Of these, 1080 consisted of adults and 3083 of nymphs. In 1771 ticks no TBPs were found. As anticipated, the most prevalent TBP detected was *B. burgdorferi* (s.l.) (21.6%), closely followed by *R. helvetica* (20.9%) and *Spiroplasma* sp. (20.1%). The TBPs *N. mikurensis* (6.0%), *B. miyamotoi* (4.1%) and *A. phagocytophilum* (3.0%) were less common, while *Babesia* (s.s.) (1.6%) and *B. microti* (0.9) had the lowest prevalence (Additional file 1). Co-infections occurred in 16.7% of ticks (Additional file 1).

### Study population

The mean age for all participants was 45.08 years of age (SD ± 19.85) with a nearly even sex distribution (2081 male and 2082 female) with no significant difference between participants that submitted a tick with a TBP and those who submitted a tick with no detected TBP. Most participants reported having completed a higher education (64.3%), while 31.7% reported completing medium education and only a fraction (3.9%) reported completing lower education. Underlying health conditions were reported by 1789 (42.9%) participants, out of which 16.6% reported more than one comorbidity. Antibiotic use was reported by 30.8% of the participants; of these, 55.7% reported having one or more complaints while in the entire cohort 43% of participants reported having one or more complaints (Additional file 1).

### Associations between TBPs and health complaints

The univariate analyses performed for each possible TBP/complaint combination showed an association with a *P* < 0.1 between 17 (out of 33 tested) complaints and at least one of the TBPs (Additional file 1).

Of the 17 complaints significant in the univariate analyses, 13 had a significant association (*P* < 0.05) with at least one TBP in the multivariate analysis (Additional file 1). Of these, 11 were positively associated with having comorbidities (represented as ‘frailty,’ Additional file 1), whereas being male was protective for six of these complaints (Additional file 1). *Babesia* (s.s.) was not significantly associated with any complaint. Below we report the associations with a *P*-value < 0.01. The complaint LB/EM was significantly associated ($P = 0.001$) with *B. burgdorferi* (s.l.) infection and negatively associated with being bitten by a nymph regardless of whether *B. burgdorferi* (s.l.) was detected in said nymph (Table 1). Facial paralysis, one of the two rarest reported complaints in this study (*n* = 10, Additional file 1) was associated ($P < 0.01$) with infection with *B. miyamotoi*, *N. mikurensis* and *R. helvetica* (Table 1). Nocturnal sweating was associated with *A. phagocytophilum* ($P < 0.01$) (Table 1).

**Table 1:** Significant associations between pathogens and self-reported complaints from multivariate analysis ($$P < 0.01)$$

Complaint	*A. phagocytophilum*	*B. miyamotoi*	*B. burgdorferi* (s.l.)	*N. mikurensis*	*R. helvetica*	Tick life stage = *N*	Frailty
LB/EM			1.75 [(1.32, 2.31), *P* = < 0.001]			0.61 [(0.46, 0.80), *P* = < 0.001]	
Facial paralysis		9.88 [(1.39, 47.40), *P* = 0.007]		10.67 [(2.14, 45.15), *P* = 0.002]	5.99 [(1.67, 24.08), *P* = 0.007]		
Nocturnal sweating	2.34 [(1.39, 3.75), *P* = 0.001]						1.35 [(1.23, 1.47), *P* = < 0.001]

The association between complaints and significant variables is given as odds ratios with their confidence intervals

### General practitioner confirmed Lyme borreliosis

Of the 244 self-reported LB/EM, 123 were verified by the patients’ GPs. Of these, 63 were confirmed as LB/EM and 60 were disproved (Additional file 1). As a sensitivity analysis for the association between developing GP-confirmed LB/EM following a tick bite by a *B. burgdorferi* (s.l.)-infected tick, the GLM was rerun using the 63 confirmed cases only, and again a significant association was found ($P < 3.97\times {10}^{-8}$) (Additional file 1).

## Discussion

Our results show significant associations between the presence of a TBP in ticks feeding on humans and self-reported symptoms. As expected, the subjective report of LB/EM was significantly associated (*P* < 0.001) with *B. burgdorferi* (s.l.) infection. Being bitten by a nymph was ostensibly protective for reported LB/EM. However, this result should be interpreted with care as this result refers to the risk of reporting LB/EM when being bitten by nymphs regardless of *B. burgdorferi* (s.l.) detection. It may thus suggest that an infected nymph gives a smaller chance of reporting LB/EM than an infected adult. The detection of the well-known association between Lyme borreliosis and the presence *B. burgdorferi* (s.l.) in GP confirmed cases suggests that our approach is capable of correctly determining associations between other self-reported complaints and the TBPs detected in ticks. Indeed, a significant association between nocturnal sweating, which is indicative for fever, and *A. phagocytophilum* was found. Remarkably, facial paralysis was associated with infection with either *B. miyamotoi, N. mikurensis* or *R. helvetica*.

Both *B. miyamotoi* and *R. helvetica* have been found to be associated with (other) neurological disorders in previous studies, but not with facial paralysis as in this study [8, 17, 18]. Facial paralysis is a well-known clinical sign of Lyme neuroborreliosis and sometimes also TBE [19–23], but has not been associated with other TBPs. As *B. miyamotoi* and *N. mikurensis* share vertebrate hosts with both TBEV and some species of *B. burgdorferi* (s.l.) [24–27], one explanation for our findings is that the ticks of these individuals were co-infected with TBEV, which was not measured in this study. Another alternative explanation is that the ticks were (co-)infected with *B. burgdorferi* (s.l.), which was not detected by our molecular assay. This could be because the bacterial load in these ticks was below the detection limit of the qPCR. In a previous prospective study, indeed some patients with EM, who were bitten by a *B. burgdorferi* (s.l.)-negative tick, were found [12]. While no statements on causality can be derived from these results, these findings give incentive to consider neurological symptoms when performing future prospective studies in patients infected by TBPs other than *B. burgdorferi* (s.l.) and TBEV [28].

The association of nocturnal sweating with *A. phagocytophilum* infection seems plausible as this symptom was hitherto linked to human granulocytic anaplasmosis (HGA) [29, 30]. While fever, the most commonly reported symptom in HGA [29, 30], was not associated with tick-bites from ticks infected with *A. phagocytophilum* in our study, it might have been experienced by participants as nocturnal sweating, which in some occasions can be caused by elevation of body temperature [31, 32]. In daily practice many patients report nocturnal sweats, but upon additional questions during a careful anamnesis, in many cases there turns out to be no need to change clothes, blankets or sheets [32].

When investigating the association between complaints and TBPs, underlying chronic health conditions should be included in the analyses since they can be associated with the complaints as well. This can be because they cause the reported symptoms or sometimes (e.g., impaired immune status [5, 8]) comorbidities might be a risk factor for infection with a TBP. Another finding that should be noted is that > 90% of the study participants completed medium or higher education, in accordance with previous publications [33, 34]. Whether their background prompted these individuals to participate in the study or biased its results is unknown. However, for a more comprehensive analysis, future studies should make an effort to include other fractions of the population.

Several limitations of our current study should be considered when assessing its results. First, the self-reported complaints in this study were mostly not corroborated by medical practitioners and merely are a subjective assessment of each participant. Nonetheless, the correctness of the self-diagnosis is independent of the TPB found and will therefore only lower the power of the GLM, and not bias our results. Second, medical complaints caused by a tick bite are mainly expected shortly after the event [35]. In our study, the questionnaires were completed 3 months after the tick bite and the complaint’s timing within this time frame remained unknown, further complicating associating the tick bite with the experienced complaints, let alone assuming causality. Third, although of relevance to our study, the use of antibiotics was not incorporated into the analysis, the reason being that the nature of the treatment (prophylactic vs. therapeutic) as well as the reason and timing were not recorded uniformly. All of the aforementioned reasons made it difficult to ascertain what kind of effect, if any, could be attributed to antibiotic use. Finally, no diagnostic assays were performed on patient-derived samples and the detection of TBPs in ticks was based on genetic fragments of the various microorganisms and not their viability or infectivity [36].

The current study provides an analysis of a large cohort that allowed us to find an association between the presence of TBPs in ticks and illness as well as diagnosed Lyme borreliosis. Future prospective longitudinal studies with appropriate control groups, better defined and objectified symptoms and signs and in which attempts are made to establish infection of the participants by means of objective clinical and laboratory findings are warranted to verify our observations.

## Supplementary Information

**Additional file 1. **Data and statistical analyses.

**Additional file 2. **Original questionnaire filled by participants at the www.tekenradar.nl website.

## Data Availability

Most of the data generated or analyzed during this study are included in this published article (and its additional information files). Other datasets used and/or analysed during the current study are available from the corresponding author on reasonable request.
