# Supplementary material for: Self-reported symptoms and health complaints associated with exposure to Ixodes ricinus-borne pathogens
Source: Parasit Vectors. 2022 Mar 18;15:93. doi: 10.1186/s13071-022-05228-4 (PMC8931963; doi:10.1186/s13071-022-05228-4)
Supplement: Supplementary file 2 — Additional file 2. Original questionnaire filled by participants at the www.tekenradar.nl website. [file 13071_2022_5228_MOESM2_ESM.docx]

Alle Tekenradarvragenlijsten - Codeboek

T=0

# B. Alle vragen per “hoofdstuk”

## Hoofdpagina = startpunt melding

**Meld een tekenbeet**

**Bent u gebeten door een teek en/of heeft u een** erythema migrans**?**

Laat het ons weten via de onderstaande buttons en doe mee aan het landelijke onderzoek naar tekenbeten en de ziekte van Lyme.

Start: Tekenbeet melden

Erythema migrans melden -> zie routing erythema migrans melding

Indien ingelogd: > ga naar Voor wie doet u deze melding

E-mail adres: ………………

Indien e-mail = onbekend -> Melden tekenbeet (TB-A)

Wachtwoord:………….

### **Voor wie doet u deze melding?**

De onderstaande personen zijn aan uw account gekoppeld. Kies de persoon voor wie u deze melding doet.

Q1. Kies een persoon:

a. Mijzelf (voornaam achternaam)

b. Anders:

Voornaam: .............

Tussenvoegsel: ............

Achternaam: ................

### Melden tekenbeet (TB-A)

De volgende vragen gaan over de tekenbeet.
Indien u meerdere tekenbeten tegelijk heeft opgelopen, kunt u dit als één tekenbeet melden.

Q2. Is deze tekenbeet (mogelijk) tijdens het werk opgelopen?

a. Ja, mijn beroep is: ...........

b. Nee

c. Weet niet

Q3. In welk type omgeving heeft u de tekenbeet opgelopen? (meerdere antwoorden mogelijk)

a. Tuin

b. Bos

c. Heide

d. Weiland

e. Stadspark

f. Duinen

g. Moerasgebied

h. Anders, namelijk: ................

i. Weet niet

Q4. Bij welke activiteit heeft u de tekenbeet opgelopen? (meerdere antwoorden mogelijk)

a. Wandelen

b. Hond uitlaten

c. Tuinieren

d. Picknicken

e. Groenbeheer

f. Spelen

g. Anders, namelijk: ..........

h. Weet niet

Q7. Weet u de locatie waar u de tekenbeet (vermoedelijk) heeft opgelopen?

a. Ja, ik weet het precies (Pop-up kaart, Fig. 1)

b. Ja, ik weet het ongeveer (Pop-up kaart, Fig. 1)

c. Ja, ik denk het te weten (Pop-up kaart, Fig. 1)

d. Nee, ik weet het niet (Geen pop-up)

**
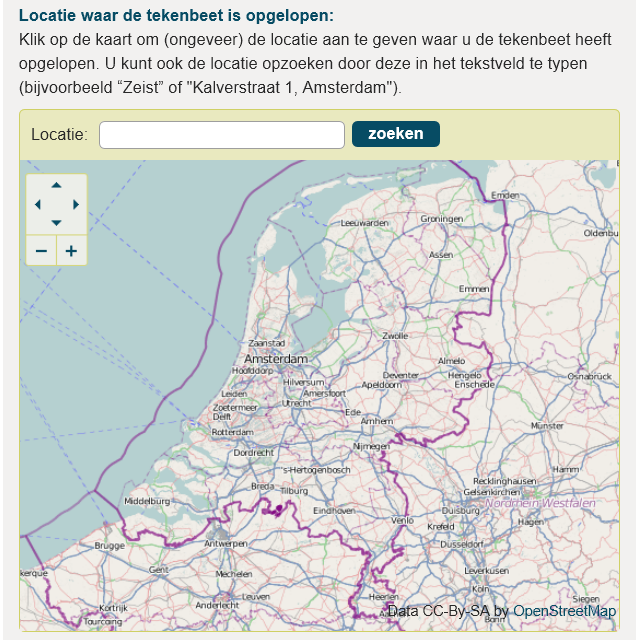
**

Fig. 1: Pop-up kaart (deelnemers worden gevraagd een markering op de kaart te plaatsen)

Q9. Zit de teek nog vast op het lichaam?

a. Ja ga naar vraag Q19

b. Nee

Q10. Heeft u de teek bewaard?

a. Ja

b. Nee kandidaat voldoet niet aan de criteria voor deelname

Q201. Wanneer heeft u de teek verwijderd?

a. Vandaag, tussen ... en .... uur

b. Gisteren, tussen .... en .... uur

c. Eergisteren, tussen .... en ..... uur

d. Eerder namelijk, ................(dag/maand/jaar) tussen ... en..... uur kandidaat voldoet niet aan criteria voor deelname

Q19. Als u deze tekenbeet niet meetelt, hoeveel tekenbeten heeft u dan in de afgelopen 5 jaar opgemerkt?

a. Geen tekenbeten ga naar vraag: Q11

b. 1 - 3 tekenbeten

c. 4 - 10 tekenbeten

d. 11 - 50 tekenbeten

e. Meer dan 50 tekenbeten

Q20. Als u deze tekenbeet niet meetelt, hoeveel tekenbeten heeft u dan in de afgelopen 3 maanden opgemerkt

a. Geen tekenbeten

b. 1 - 3 tekenbeten kandidaat voldoet niet aan de criteria

c. 4 - 10 tekenbeten kandidaat voldoet niet aan de criteria

d. 11 - 50 tekenbeten kandidaat voldoet niet aan de criteria

e. Meer dan 50 tekenbeten kandidaat voldoet niet aan de criteria

Kandidaten die niet aan de criteria voor deelname voldoen krijgen onderstaande vragen niet te zien, zij krijgen alleen nog hoofdstuk Tekenbeten algemeen (TB-D) te zien om in te vullen.

Ook mensen die reeds meedoen krijgen alleen nog vragenlijst Tekenbeten algemeen (TB-D) te zien!!!!!

### Uitnodiging onderzoeksdeelname

Mensen met een tekenbeet wordt gevraagd mee te doen aan onderzoek naar tekenbeten en de ziekte van Lyme. Voor het onderzoek vragen wij u de teek naar het RIVM te sturen en om anderhalf jaar lang elke drie maanden een korte vragenlijst in te vullen op Tekenradar.nl. Binnen 10 maanden wordt dan getest of de teek besmet was met de Borrelia bacterie die de ziekte van Lyme kan veroorzaken.

Q11. Wilt u mee doen aan het onderzoek van het RIVM naar tekenbeten en de ziekte van Lyme?

a. Ja

b. Nee -> geen deelname (-> TB-D)

Met onderstaande vragen wordt bepaald of u kunt meedoen aan het onderzoek.

Q202. Voor deelname is het belangrijk dat u ouder bent dan 8 jaar en dat u niet zwanger bent. Voldoet u aan deze criteria?

a. Ja, ik ben ouder dan 8 jaar en ik ben niet zwanger, mijn leeftijd is ……

b. Nee, ik ben jonger dan 8 jaar of ik ben zwanger kandidaat voldoet niet aan criteria voor deelname

Q203. Voor dit onderzoek kunt u gevraagd worden om antibiotica te gebruiken. Heeft u ooit een allergische reactie gehad door antibiotica?

a. Ja

b. Nee >naar volgend hoofdstuk

Q204. Door welke soort antibiotica kreeg u deze allergische reactie?

a. Doxycycline of andere tetracyclines -> kandidaat voldoet niet aan criteria voor deelname

b. Andere antibiotica soort (en) (bijvoorbeeld penicillines)

Kandidaten die niet aan de criteria voor deelname voldoen krijgen onderstaande hoofdstukken niet te zien, zij krijgen alleen nog hoofdstuk Tekenbeten algemeen (TB-D) te zien om in te vullen.

Ook mensen die reeds meedoen krijgen alleen nog vragenlijst Tekenbeten algemeen (TB-D) te zien!!!!!

### Registreren?

**Ik heb geen account**

Met een account kunt u:

•Tekenbeten en erythema migrans(?) registreren voor uzelf en uw gezinsleden

•Inzicht krijgen in uw geregistreerde tekenbeten en erythema migrans(?)

•Bijdragen aan onderzoek naar tekenbeten en de ziekte van Lyme

U kunt eventueel bij een volgende registratie alsnog een account aanmaken.

Account aanmaken (klikbaar) -> account aanmaken

**Ik heb al een account**

E-mailadres:………….

Wachtwoord:……………….

Inloggen (klikbaar)-> voor wie doet u deze melding

Wachtwoord vergeten? (klikbaar)> vul hier uw email in -> email verstuurd

### Account aanmaken

Account aanmaken

Voornaam:…………………………

Tussenvoegsel:…………………

Achternaam:……………………..

E-mailadres:…………………

Wachtwoord:……………..

Bevestig wachtwoord:……………….

### Voor wie doet u deze melding?

De onderstaande personen zijn aan uw account gekoppeld. Kies de persoon voor wie u deze melding doet.

Q1. Kies een persoon :

Mijzelf (naam prefix achternaam)

Mijn kind

Etc.

Anders

Voornaam:……………………..

Tussenvoegsel:……………….

Achternaam:…………………….

### Informatie over het onderzoek

(graag alleen kopjes laten zien die klikbaar zijn, en de tekst alleen na aanklikken van kopje ...o.i.d. afweging maken tussen wat nog goed op een pagina past, maar dat het wel heel duidelijk is dat men de informatie ook hier te zien kan krijgen als men dat wil)

Deelnemers worden random verdeeld over de 2 onderzoeksgroepen. Deelnemers blijven voor de periode van de studie (= 1,5 jaar) in dezelfde onderzoeksgroep zitten. Daarnaast zullen ook hun eventuele partners indien zij onder hetzelfde account een melding doen in deze zelfde groep ingedeeld worden.

De eerste groep krijgt een eenmalige dosis antibiotica voorgeschreven, zij krijgen hiervoor een e-mail toegestuurd met informatie voor hun huisarts, welke via een button ook direct te printen is.

De tweede onderzoeksgroep krijgt geen AB, maar wordt wel gevraagd de vragenlijsten in te vullen en teek op te sturen. Hiervoor moet een variabele “Overzoeksgroep” aangemaakt worden. Daarnaast moet er een tweede variabele komen waarin gecodeerd wordt of de deelnemer antibiotica geslikt heeft of niet (In principe zou dit gelijk moeten liggen met de onderzoeksgroep, maar realiteit is waarschijnlijk anders)= “ABgroep”

U heeft zojuist een tekenbeet gemeld op Tekenradar.nl. Wij willen u vragen om mee te doen aan een onderzoek naar preventieve behandeling met antibiotica na een tekenbeet. Een preventieve behandeling is een behandeling **ter voorkoming** van een ziekte, in dit geval de ziekte van Lyme.

De kans op de ziekte van Lyme na een tekenbeet is klein (ongeveer 2 op de 100). Als iemand ziek wordt dan geneest hij meestal na behandeling. Met dit onderzoek willen wij bepalen of **preventief** behandelen met antibiotica kort na een tekenbeet leidt tot minder mensen met de ziekte van Lyme. En als dat zo is, weegt dat dan op tegen de nadelen van preventief behandelen? Want preventief behandelen houdt in dat alle mensen na een tekenbeet antibiotica krijgen, ook al is de kans op het krijgen van de ziekte klein. En dat betekent veel onnodig gebruik van antibiotica. Een ander nadeel is dat er bijwerkingen door de antibiotica kunnen optreden.

Dit onderzoek willen wij uitvoeren bij 2500 mensen. Bij de start van het onderzoek vragen wij de helft van de deelnemers om één keer preventieve antibiotica in te nemen – via de eigen huisarts en binnen 72 uur na verwijderen van de teek – en de andere helft om dat niet te doen. Tijdens het onderzoek vullen de deelnemers vragenlijsten over hun gezondheid in op Tekenradar.nl. Vervolgens zal gekeken worden hoeveel mensen in de beide groepen de ziekte van Lyme ontwikkelen en/of bijverschijnselen door de antibiotica melden.

We hopen op uw medewerking, zodat in de toekomst beter advies gegeven kan worden over preventieve antibiotica na een tekenbeet. Hieronder vindt u meer informatie over het onderzoek en de ziekte van Lyme en kunt u een toestemmingsformulier invullen als u mee wilt doen. Voor kinderen die aan het onderzoek willen deelnemen vragen wij de ouders/verzorgers om de informatie hieronder mee te lezen.

Bij voorbaat onze hartelijke dank!

Met vriendelijke groet,

Dr. Wilfrid van Pelt

Afdelingshoofd

RIVM-Centrum voor Infectieziektebestrijding

Vanaf hier alleen de kopjes laten zien op de webpagina, met mogelijkheid tot aanklikken om de hele tekst uit te laten klappen

**Wat is de ziekte van Lyme?**

De ziekte van Lyme wordt veroorzaakt door een bacterie die via de beet van een teek in uw lichaam komt. Het eerste signaal is vaak een rode ringvormige uitslag (een “erythema migrans”) rond de plaats van de tekenbeet, die langzaam groter wordt. De ziekte van Lyme kan ook algemene klachten veroorzaken als hoofdpijn, koorts of gewrichtspijn. Mensen met een erythema migrans of gezondheidsklachten na een tekenbeet doen er goed aan om naar de huisarts te gaan, zodat ze behandeld kunnen worden met een volledige antibioticakuur. Als de ziekte van Lyme niet behandeld wordt, kan het erger worden en ernstige klachten geven zoals uitstralende pijn in armen of benen, uitval van gezichtsspieren, hartritmestoornissen, gewrichtsklachten en chronische huidaandoeningen.

**Wat is het doel van het onderzoek?**

Doel van het onderzoek is bepalen of een preventieve behandeling met het antibioticum *doxycycline* na een tekenbeet werkt om ziekte van Lyme te voorkómen. Dit antibioticum (in 2 tabletten) hoeft maar één keer ingenomen te worden. Zonder preventieve antibiotica is de kans op de ziekte van Lyme na een tekenbeet al relatief klein: ongeveer 2 op de 100 mensen krijgen ziekteverschijnselen die meestal verdwijnen na behandeling. Bij preventief behandelen met antibiotica zouden dus veel mensen onnodig behandeld worden, met risico op bijverschijnselen door de antibiotica. Of de voordelen van preventieve antibiotica (minder ziekte van Lyme) groter zijn dan de nadelen (onnodig gebruik van antibiotica, bijwerkingen van de antibiotica) is voor Nederland nog niet onderzocht. Daarom zal in dit onderzoek de helft van de deelnemers gevraagd worden om één keer preventieve antibiotica te nemen – via de eigen huisarts – en de andere helft om dat niet te doen. Vervolgens zal gekeken worden hoeveel mensen in de beide groepen de ziekte van Lyme krijgen en er wordt gekeken of de antibiotica bijwerkingen geven. Dit onderzoek willen wij uitvoeren bij 2500 mensen.

**Wie kan meedoen aan het onderzoek?**

U kunt meedoen aan dit onderzoek als u voldoet aan deze voorwaarden:

-U bent niet zwanger

-U heeft de teek korter dan 3 dagen (72 uur) geleden verwijderd

-U heeft de teek bewaard (of hij zit nog vast), en stuurt hem op naar het RIVM

-U heeft in de 3 maanden voor deze tekenbeet geen andere tekenbeten gehad

Kinderen die meedoen aan het onderzoek moeten 8 jaar of ouder zijn en de ouders/verzorgers moeten toestemming voor deelname geven. Wij vragen de ouders/verzorgers ook om mee te helpen met het opsturen van de teek en het invullen van de vragenlijsten.

**Wat vragen wij van u?**

U meldt de tekenbeet en stuurt de teek op naar het RIVM volgens de aanwijzingen op de website Tekenradar.nl. De teek die u opstuurt wordt getest op de bacterie die de ziekte van Lyme kan veroorzaken.

U vult het toestemmingsformulier op Tekenradar.nl in. U ontvangt daarna een kopie van het door u ingevulde formulier per e-mail. Na printen en ondertekening stuurt u het formulier op naar het aangegeven adres.

Na het invullen van de toestemmingsverklaring wordt u door middel van toeval ingedeeld in de behandelgroep of in de niet-behandelgroep.

Als u in de **behandelgroep** komt vragen wij u om zo snel mogelijk uw eigen huisarts te bezoeken, om 2 tabletten preventieve antibiotica voorgeschreven te krijgen. Dat moet binnen 3 dagen (dus 72 uur) na verwijderen van de teek worden ingenomen – voor een tekenbeet in het weekend kunt u dus na het weekend naar uw eigen huisarts gaan. Alle huisartsen in Nederland hebben van ons informatie ontvangen over dit onderzoek en zijn gevraagd om mee te werken. Wij sturen u per e-mail een brief voor uw huisarts waarin staat dat u meedoet aan dit onderzoek. Wij vragen u deze brief uit te printen en mee te nemen naar uw huisarts. Met uw huisarts kunt u vervolgens bespreken of u inderdaad de preventieve antibiotica kunt nemen. De antibiotica kunt u met het recept van uw huisarts ophalen bij een apotheek.
Als u in de **niet-behandelgroep** komt vragen we u om geen antibiotica te halen bij de huisarts voor direct na de tekenbeet.

**Alle** deelnemers worden verzocht om op een aantal momenten (bij de start van de studie, na 1 week, na 1 maand en na 3 maanden, en daarna elke 3 maanden) een vragenlijst in te vullen. Deelname aan het onderzoek duurt anderhalf jaar. De vragenlijsten staan op de website Tekenradar.nl. De vragen gaan over uw gezondheid, eventuele bijwerkingen van de antibiotica en eventuele ziekteverschijnselen door de ziekte van Lyme. U ontvangt steeds een uitnodiging voor de vragenlijsten per email.

Als u klachten krijgt die wijzen op de ziekte van Lyme of het gevolg zijn van de antibiotica, dan dient u contact op te nemen met uw huisarts. Wij vragen uw toestemming om de gegevens van zo’n bezoek op te vragen bij uw arts.

**Wat is de standaardbehandeling na een tekenbeet?**

In Nederland is de standaardbehandeling na een tekenbeet om af te wachten en alleen de mensen die gezondheidsklachten ontwikkelen met antibiotica te behandelen.

**Wat zijn de voor- en nadelen van deelname aan dit onderzoek?**

Als u bent ingedeeld in de **niet-behandelgroep**, dan is er geen nadeel van deelname aan dit onderzoek, omdat het in Nederland gebruikelijk is om mensen direct na een tekenbeet geen preventieve antibiotica te geven.

Als u bent ingedeeld in de **behandelgroep**, dan heeft u waarschijnlijk een kleinere kans om de ziekte van Lyme te krijgen, maar heeft u ook een kleine kans op bijwerkingen van de antibiotica.

**Welke bijwerkingen kunt u verwachten? (als u in de behandelgroep wordt ingedeeld)**

Zoals alle geneesmiddelen kan de antibiotica soms bijwerkingen veroorzaken. De meest voorkomende bijwerkingen zijn maagdarmklachten. Ook maakt de gebruikte antibiotica (doxycycline) u gevoeliger voor verbranding door de zon. Daarom kunt u de dag dat u de antibiotica hebt ingenomen fel zonlicht beter vermijden. Als u bijwerkingen krijgt kunt u dat melden in de vragenlijsten.

**Wat gebeurt er als u niet wilt deelnemen aan dit onderzoek?**

Deelname is vrijwillig. Als u besluit niet mee te doen, hoeft u verder niets te doen. U hoeft ook de verwijderde teek niet op te sturen. Als u wel meedoet, kunt u zich altijd bedenken en toch stoppen. Ook tijdens het onderzoek.

**Hoe hoor ik over de uitkomsten van dit onderzoek?**

Alleen de teken van de deelnemers die de vragenlijsten blijven invullen worden getest. U ontvangt binnen 10 maanden bericht per email of uw teek besmet was met de bacterie die de ziekte van Lyme kan veroorzaken, tenzij u tevoren heeft aangegeven dat u de uitslag niet wilt weten.

Na afloop van het onderzoek eind 2016 ontvangen alle deelnemers een email met de uitkomsten van het onderzoek.

**Wat gebeurt er met uw gegevens?**

Uw gegevens worden vertrouwelijk en zorgvuldig behandeld. Naast de onderzoeksmedewerkers kunnen alleen medewerkers van de Inspectie voor de Gezondheidszorg, leden van de medisch-ethische toetsingscommissie of andere bevoegde personen inzage krijgen in het onderzoeksdossier. Al uw persoonsgegevens worden gecodeerd verwerkt, zodat in de verslaglegging en publicaties niets meer terug te voeren is op u persoonlijk. Alle onderzoeksgegevens worden, in overeenstemming met de wettelijke bepalingen, gedurende 15 jaar bewaard en daarna vernietigd. Mogelijk kunnen we uw gegevens in de toekomst gebruiken voor een ander onderzoek. Als u dat niet wilt, respecteren we dat vanzelfsprekend. U kunt uw weigering op het toestemmingsformulier schriftelijk vastleggen. Als u daar geen bezwaar tegen hebt, kunt u dat op het toestemmingsformulier ook aangeven. We zullen u, wanneer dat andere onderzoek uitgevoerd zal gaan worden, daarover informeren. U kunt dan alsnog aangeven of uw gegevens daar wel of niet voor mogen worden gebruikt. We benaderen u pas nadat de medisch-ethische toetsingscommissie die dit onderzoek goedkeurde ook dat andere onderzoek heeft goedgekeurd.

**Bent u verzekerd wanneer u aan dit onderzoek meedoet?**

Het is in Nederland verplicht om bij ieder onderzoek een (standaard) verzekeringsbijlage ter informatie mee te sturen aan deelnemers. Het RIVM hoeft als overheid geen verzekeringen af te sluiten voor deelnemers aan het onderzoek. Als u toch schade oploopt als gevolg van het onderzoek kunt u dat bij ons melden.

Het RIVM zal dan handelen volgens de normale verzekeringsvoorwaarden, zie hiervoor de verzekeringsbijlage waarop u de verzekerde bedragen, de uitzonderingen en de contactgegevens van het RIVM vindt. Dit geldt voor schade die naar voren komt tijdens het onderzoek, of binnen vier jaar na het einde van het onderzoek.

**Welke medisch-ethische toetsingscommissie heeft dit onderzoek goedgekeurd?**

In Nederland bestaan strenge wettelijke regels voor zogenoemd mensgebonden onderzoek. De regels staan in de Wet medisch-wetenschappelijk onderzoek met mensen. Een onderzoek mag pas beginnen als een speciale toetsingscommissie het onderzoek heeft goedgekeurd. Dit onderzoek is goedgekeurd door de medisch-ethische toetsingscommissie (METC) Noord-Holland.

**Verzekeringsbijlage**

Voor de deelnemers aan dit onderzoek draagt het RIVM zorg voor schadeloosstelling van deelnemers aan dit onderzoek, volgens de Wet Medisch-Wetenschappelijk Onderzoek met mensen (WMO), verzekeringsbesluit 2003. Het RIVM dekt schade door dood of letsel die het gevolg is van deelname aan het onderzoek en die zich gedurende de deelname aan het onderzoek openbaart, of binnen vier jaar na beëindiging van de deelname aan het onderzoek. De schade wordt geacht zich te hebben geopenbaard wanneer deze bij het RIVM is gemeld.

In geval van schade kunt u contact opnemen met het RIVM:
RIVM
Postbus 1
3720 BA Bilthoven
Contactpersoon: Dhr. A.N. van der Zande
Tel. 030 - 274 2576
E-mail Andre.van.der.zande@rivm.nl

Het RIVM biedt een maximum dekking van € 450.000 per proefpersoon en

€ 3.500.000 voor het gehele onderzoek. De dekking van specifieke schades en kosten is verder tot bepaalde bedragen beperkt. Deze vindt u in het Besluit verplichte verzekering bij medisch-wetenschappelijk onderzoek met mensen. Informatie hierover kunt u vinden op de website van de Centrale Commissie Mensgebonden Onderzoek: [www.ccmo.nl.](http://www.ccmo.nl/)

Voor deze verzekering gelden een aantal uitsluitingen. De verzekering dekt niet:

schade waarvan op grond van de aard van het onderzoek zeker of nagenoeg zeker was dat deze zich zou voordoen;

schade aan de gezondheid die ook zou zijn ontstaan indien u niet aan het onderzoek had deelgenomen;

schade die het gevolg is van het niet of niet volledig nakomen van aanwijzingen of instructies;

schade aan nakomelingen, als gevolg van een nadelige inwerking van het onderzoek op u of uw nakomeling;

bij onderzoek naar bestaande behandelmethoden: schade die het gevolg is van één van deze behandelmethoden;

bij onderzoek naar de behandeling van specifieke gezondheidsproblemen: schade die het gevolg is van het niet verbeteren of van het verslechteren van deze gezondheidsproblemen.

**Wilt u meer weten over het preventieve antibiotica-onderzoek?**

Als u na het lezen van deze informatie nog vragen heeft over het onderzoek neem dan tijdens kantooruren telefonisch contact op met de betrokken onderzoekers Margriet Harms (030-2742273), of Kees van den Wijngaard (030-2742910). Als u voor of tijdens de studie vragen heeft die u liever niet aan de onderzoekers stelt, kunt u contact opnemen met de onafhankelijke arts Hans van Vliet (medisch adviseur, telefoonnummer 030-2744376).

Direct onder de informatie over het onderzoek worden de vragen voor de toestemmingsverklaring getoond:

Koptekst:

Indien leeftijd <12 (gegeven door Q202a)

Vul hieronder gegevens van uw kind in

Indien leeftijd 12-17

Indien leeftijd 18+

Vul hieronder uw gegevens in:

Q146. Voornaam: …… (vooringevuld)

Q147: Achternaam: ……… (vooringevuld)

Q148: Straat: ……..

Q149: Postcode……

Q150: Plaats…….

Q152: Geboortedatum…….

Q26: Wat is uw geslacht? (q26 voor deelnemers wordt hier gesteld, voor anonieme meldingen zit

a.Man deze in TB-D)

b. Vrouw

Q145: Wilt u binnen 10 maanden per email de uitslag krijgen van uw geteste teek?

1. Ja
2. Nee

Q144: Ik geef a. wel / b. geen toestemming om in de toekomst mijn gegevens te gebruiken voor nader onderzoek of vervolgonderzoek en daarvoor benaderd te worden.

### Toestemmingsverklaring

Op basis van Q202a wordt hier de juiste toestemmingsverklaring getoond.

(3 versies: 8 t/m 11 jaar

12 t/m 17 jaar

18 jaar en ouder)

*Voor deelnemers van 8 t/m 11 jaar: deze toestemmingsverklaring dient ondertekend te worden door beide ouders / verzorgers van het kind dat meedoet aan het onderzoek.*

- Ik verklaar de informatiebrief over het onderzoek te hebben gelezen. Ik begrijp de informatie. Ik heb de gelegenheid gehad om aanvullende vragen te stellen. Deze vragen zijn naar tevredenheid beantwoord. Ik heb voldoende tijd gehad om over deelname na te denken.
- Ik begrijp dat deelnemen aan het onderzoek het volgende inhoudt:

1. Ik meld een tekenbeet bij mijn kind op Tekenradar.nl en stuur de teek op naar het RIVM volgens de instructies op de Tekenradar.nl site.
2. Mijn kind bezoekt zo snel mogelijk de huisarts voor het nemen van preventieve antibiotica, als hij/zij in de behandelgroep wordt ingedeeld.
3. Ik vul gedurende anderhalf jaar samen met mijn kind de periodieke vragenlijsten in op Tekenradar.nl

- Ik weet dat deelname geheel vrijwillig is en dat afzien van deelname geen gevolgen heeft.
- Ik weet dat medewerkers van de Inspectie voor de Gezondheidszorg, leden van de medisch-ethische toetsingscommissie of andere bevoegde personen inzage kunnen krijgen in het onderzoeksdossier om onderzoeksgegevens en -procedures te verifiëren. Alle gegevens zullen vertrouwelijk behandeld worden.
- Ik geef toestemming om de gegevens te verwerken voor de doelen zoals beschreven in de informatiebrief. Ik geef toestemming om de gecodeerde onderzoeksgegevens gedurende 15 jaar te bewaren.
- Ik geef toestemming voor het opvragen van relevante medische gegevens bij de huisarts / specialist / behandelend arts tijdens de onderzoeksperiode.

_ Onderstaande alleen in papieren versie _________________________________________________________________________

Ik geef a▢ WEL toestemming b▢ GEEN toestemming

om in de toekomst mijn gegevens te gebruiken voor nader onderzoek of vervolgonderzoek en daarvoor benaderd te worden.

Wilt u binnen 10 maanden per email de uitslag krijgen van uw geteste teek?

▢ JA ▢ NEE

*Vul hieronder de gegevens van uw kind in:vooringevuld met de boven ingevulde gegevens*

**VOORLETTERS:………….ACHTERNAAM**:………………………………………..

**STRAAT:…………………………………………………………. HUISNUMMER:……..**

**POSTCODE:…………….. PLAATS:……………………………………………….**

Geboortedatum: |__|__| - |__|__| - |__|__|__|__| (dd/mm/jjjj)

**HANDTEKENING** beide ouders/verzorgers:

…………………………………….. ……………………………………..

Plaats: ……………….. Datum: |__|__| - |__|__| - 20|__|__| (dd/mm/jjjj)

*Stuur het formulier na printen en ondertekening op naar: RIVM, Tekenradar (pb 75), Antwoordnummer 3266,3720 VB BILTHOVEN (postzegel niet nodig)*

__________________________________________________________________________

*In te vullen door de RIVM-onderzoeksmedewerker:*

Ik verklaar dat bovengenoemde deelnemer volledig is geïnformeerd over het onderzoek.

Handtekening: …………………………………………..

Naam onderzoeksmedewerker:…………………………………………..

Datum: |__|__| - |__|__| - 20|__|__| (dd/mm/jjjj)

QR – code voor snelle verwerking van de binnen komende teken

*Plak hier uw teek*

xxxxTRxxxx

*Voor deelnemers van 12 t/m 17 jaar: deze toestemmingsverklaring dient ondertekend te worden door de deelnemer EN door beide ouders / verzorgers van de deelnemer aan het onderzoek.*

- Ik verklaar de informatiebrief over het onderzoek te hebben gelezen. Ik begrijp de informatie. Ik heb de gelegenheid gehad om aanvullende vragen te stellen. Deze vragen zijn naar tevredenheid beantwoord. Ik heb voldoende tijd gehad om over deelname na te denken.
- Ik begrijp dat deelnemen aan het onderzoek het volgende inhoudt:

1. Ik meld een tekenbeet op Tekenradar.nl en stuur de teek op naar het RIVM volgens de instructies op de Tekenradar.nl site.
2. Ik bezoek mijn huisarts zo snel mogelijk voor het nemen van preventieve antibiotica, als ik in de behandelgroep wordt ingedeeld.
3. Ik vul gedurende anderhalf jaar de periodieke vragenlijsten in op Tekenradar.nl

- Ik weet dat deelname geheel vrijwillig is en dat afzien van deelname geen gevolgen heeft.
- Ik weet dat medewerkers van de Inspectie voor de Gezondheidszorg, leden van de medisch-ethische toetsingscommissie of andere bevoegde personen inzage kunnen krijgen in het onderzoeksdossier om onderzoeksgegevens en -procedures te verifiëren. Alle gegevens zullen vertrouwelijk behandeld worden.
- Ik geef toestemming om de gegevens te verwerken voor de doelen zoals beschreven in de informatiebrief. Ik geef toestemming om de gecodeerde onderzoeksgegevens gedurende 15 jaar te bewaren.
- Ik geef toestemming voor het opvragen van relevante medische gegevens bij mijn huisarts / specialist / behandelend arts tijdens de onderzoeksperiode.

_ Onderstaande alleen in papieren versie _________________________________________________________________________

TV1Ik geef a▢ WEL toestemming b▢ GEEN toestemming

om in de toekomst mijn gegevens te gebruiken voor nader onderzoek of vervolgonderzoek en daarvoor benaderd te worden.

TV2Wilt u binnen 10 maanden per email de uitslag krijgen van uw geteste teek?

a▢ JA b▢ NEE

*Vul hieronder uw gegevens in: vooringevuld met de boven ingevulde gegevens*

**TV3VOORLETTERS:…………..lastnameACHTERNAAM**:………………………………………..

**streetSTRAAT:…………………………………………………………. HUISNUMMER:……..**

**postalcodePOSTCODE:…………….. cityPLAATS:……………………………………………….**

TV4Geboortedatum: |__|__| - |__|__| - |__|__|__|__| (dd/mm/jjjj)

**HANDTEKENING** deelnemer: EN **HANDTEKENING** beide ouders/verzorgers:

…………………………………….. ……………………………………………………………

Plaats: ……………….. Datum: |__|__| - |__|__| - 20|__|__| (dd/mm/jjjj)

*Stuur het formulier na printen en ondertekening op naar: RIVM, Tekenradar (pb 75), Antwoordnummer 3266,*

*3720 VB BILTHOVEN (postzegel niet nodig)*

__________________________________________________________________________

*In te vullen door de RIVM-onderzoeksmedewerker:*

Ik verklaar dat bovengenoemde deelnemer volledig is geïnformeerd over het onderzoek.

Handtekening: …………………………………………..

Naam onderzoeksmedewerker:…………………………………………..

Datum: |__|__| - |__|__| - 20|__|__| (dd/mm/jjjj)

QR – code voor snelle verwerking van de binnen komende teken

*Plak hier uw teek*

xxxxTRxxxx

*Voor deelnemers van 18 jaar en ouder*

- Ik verklaar de informatiebrief over het onderzoek te hebben gelezen. Ik begrijp de informatie. Ik heb de gelegenheid gehad om aanvullende vragen te stellen. Deze vragen zijn naar tevredenheid beantwoord. Ik heb voldoende tijd gehad om over deelname na te denken.
- Ik begrijp dat deelnemen aan het onderzoek het volgende inhoudt:

1. Ik meld een tekenbeet op Tekenradar.nl en stuur de teek op naar het RIVM volgens de instructies op de Tekenradar.nl site.
2. Ik bezoek mijn huisarts zo snel mogelijk voor het nemen van preventieve antibiotica, als ik in de behandelgroep wordt ingedeeld.
3. Ik vul gedurende anderhalf jaar de periodieke vragenlijsten in op Tekenradar.nl

- Ik weet dat deelname geheel vrijwillig is en dat afzien van deelname geen gevolgen heeft.
- Ik weet dat medewerkers van de Inspectie voor de Gezondheidszorg, leden van de medisch-ethische toetsingscommissie of andere bevoegde personen inzage kunnen krijgen in het onderzoeksdossier om onderzoeksgegevens en -procedures te verifiëren. Alle gegevens zullen vertrouwelijk behandeld worden.
- Ik geef toestemming om de gegevens te verwerken voor de doelen zoals beschreven in de informatiebrief. Ik geef toestemming om de gecodeerde onderzoeksgegevens gedurende 15 jaar te bewaren.
- Ik geef toestemming voor het opvragen van relevante medische gegevens bij mijn huisarts / specialist / behandelend arts tijdens de onderzoeksperiode.

Onderstaande alleen in papieren versie __________________________________________________________________________

TV1Ik geef ▢ WEL toestemming ▢ GEEN toestemming

om in de toekomst mijn gegevens te gebruiken voor nader onderzoek of vervolgonderzoek en daarvoor benaderd te worden.

TV2Wilt u binnen 10 maanden per email de uitslag krijgen van uw geteste teek?

▢ JA ▢ NEE

*Vul hieronder uw gegevens in: vooringevuld met de boven ingevulde gegevens*

**TV3VOORLETTERS:…………..lastnameACHTERNAAM**:………………………………………..

**streetSTRAAT:…………………………………………………………. HUISNUMMER:……..**

**postalcodePOSTCODE:…………….. cityPLAATS:……………………………………………….**

TV4Geboortedatum: |__|__| - |__|__| - |__|__|__|__| (dd/mm/jjjj)

**HANDTEKENING** deelnemer: …………………………………………..

Plaats: ……………….. Datum: |__|__| - |__|__| - 20|__|__| (dd/mm/jjjj)

*Stuur het formulier na printen en ondertekening op naar: RIVM, Tekenradar (pb 75), Antwoordnummer 3266,*

*3720 VB BILTHOVEN (postzegel niet nodig)*

__________________________________________________________________________

*In te vullen door de RIVM-onderzoeksmedewerker:*

Ik verklaar dat bovengenoemde deelnemer volledig is geïnformeerd over het onderzoek.

Handtekening: …………………………………………..

Naam onderzoeksmedewerker:…………………………………………..

Datum: |__|__| - |__|__| - 20|__|__| (dd/mm/jjjj)

QR – code voor snelle verwerking van de binnen komende teken

*Plak hier uw teek*

xxxxTRxxxx

Onder de toestemmingsverklaring:

Q153: a. Ik ga akkoord met bovenstaande toestemmigsverklaring (klikbaar) >Accoord

b. Ik ga niet akkoord met bovenstaande toestemmingsverklaring (klikbaar) >Niet accoord

Indien men aanklinkt dat men accoord gaat:

#### Accoord

[randomisatie vindt plaats op moment van accoord gaan, zie onderzoeksprotocol]

Voor behandelgroep:

Dank voor uw deelname aan de studie. U bent ingedeeld in de **behandelgroep**.

Klik hier om de toestemmingsverklaring direct te printen en te ondertekenen. **LET OP: klik daarna op “volgende” onderaan dit scherm en vul de rest van de vragen in!** Stuur de toestemmingsverklaring samen met de teek vandaag nog op naar onderstaand antwoordnummer (geen postzegel nodig).

RIVM

Tekenradar (pb 75)

Antwoordnummer 3266

3720 VB BILTHOVEN

Er wordt aan het einde van deze vragenlijst een kopie van uw toestemmingsverklaring naar uw e-mail adres gestuurd, ook ontvangt u dan nogmaals alle informatie van deze studie.

Instructies over hoe u de teek het beste kunt versturen krijgt u aan het einde van deze vragenlijst.

Ook vragen wij u zo snel mogelijk een afspraak te maken met uw huisarts. Nadat u de vragenlijst volledig hebt ingevuld ontvangt u van tekenradar een e-mail met hierin de informatie voor de huisarts. Ook deze informatie moet u uitprinten meenemen bij de afspraak. Hierin staat namelijk welke richtlijnen de huisarts moet volgen voor behandeling en welk medicijn hij moet voorschrijven.

Voor niet-behandelgroep:

Dank voor uw deelname aan de studie. U bent ingedeeld in de **niet-behandelgroep**.

Klik hier om de toestemmingsverklaring direct te printen en te ondertekenen. **LET OP: klik daarna op “volgende” onderaan dit scherm en vul de rest van de vragen in!** Stuur uw toestemmingsverklaring saen met uw teek vandaag nog op naar onderstaand antwoordnummer (geen postzegel nodig).

RIVM

Tekenradar (pb 75)

Antwoordnummer 3266

3720 VB BILTHOVEN

Instructies over hoe u de teek het beste kunt versturen krijgt u aan het einde van de vragenlijst te zien.

Er wordt aan het einde van deze vragenlijst een kopie van uw toestemmingsverklaring naar uw e-mail adres gestuurd, ook ontvangt u dan nogmaals alle informatie van deze studie.

Indien men heeft aangeklikt niet accoord te gaan met de toestemmingsverklaring

#### Niet accoord

U gaat niet akkoord met de toestemmingsverklaring en wilt niet meedoen aan het onderzoek.

Wij stellen het op prijs als u hieronder de reden aangeeft:

Q154: ...........................................

Wij testen alleen de teken van mensen die deelnemen aan de studie en de vervolgvragenlijsten invullen. Omdat u heeft aangegeven niet mee te willen doen aan de studie hoeft u uw teek dus ook niet aan het RIVM op te sturen, voor onderzoek naar de Borrelia bacterie die de ziekte van Lyme kan veroorzaken. Wel zouden wij u graag nog een aantal aanvullende vragen stellen omtrent uw melding van een tekenbeet.

Deze mensen worden doorgeleid naar Tekenbeten algemeen (TB-D)zonder deelname aan de studie (gelijk aan anonieme melding)

### Achtergrond (TB-C)

Wordt voor iedere deelnemer slechts 1x gevraagd

Achtergrond

Tekenbeet melden voor: mijzelf (voornaam achternaam)

De volgende vragen gebruiken wij alleen om te bekijken of de deelnemers aan het onderzoek een goede afspiegeling zijn van de Nederlandse samenleving.

Q27. Wat is uw hoogst voltooide opleiding?

a. Geen voltooide opleiding (lager onderwijs niet afgemaakt)

b. Lager onderwijs (basisschool, speciaal basisonderwijs)

c. Lager of voorbereidend beroepsonderwijs (zoals LTS, LEAO, LHNO, VMBO)

d. Middelbaar algemeen voortgezet onderwijs (zoals MAVO, (M)ULO, MBO-kort, VMBO-t)

e. Middelbaar beroepsonderwijs en beroepsbegeleidend onderwijs (zoals MBO-lang, MTS, MEAO, BOL, BBL, INAS)

f. Hoger algemeen en voorbereidend wetenschappelijk onderwijs (zoals HAVO, VWO, Atheneum, Gymnasium, HBS,MMS)

g. Hoger beroepsonderwijs (zoals HBO, HTS, HEAO, kandidaatswetenschappelijk onderwijs)

h. Wetenschappelijk onderwijs (universiteit)

### Tekenbeten algemeen (TB-D)

Tekenbeet melden voor: mijzelf (voornaam achternaam)

Indien onderzoeksdeelnemer: ga naar: Q12

Voor anonieme melding toevoegen Q5+Q6:

Q5. Wat is uw geboortejaar: ………….

Q6. Wat is uw woonplaats of postcode:………………………

Q12. Wie heeft de teek verwijderd? (meerdere antwoorden mogelijk)

a. De teek viel er vanzelf af

b. Huisarts

c. Uzelf

d. Iemand anders, namelijk: .......................

e. Onbewust / per ongeluk verwijderd (bijvoorbeeld door te krabben of bij het afdrogen)

Q205. Wanneer heeft u de tekenbeet (vermoedelijk) opgelopen?

a. Vandaag, tussen ... en ...uur

b. Gisteren, tussen ... en ... uur

c. Eergisteren, tussen ... en ... uur

d. Eerder, op ....... (dag/maand/jaar) tussen... en ...uur

Q13. Hoe lang heeft de teek in de huid vastgezeten?

a. Korter dan 12 uur, namelijk ....... uur

b. 12 - 24 uur, namelijk …… uur

c. Langer dan 24 uur, namelijk ...... dagen/uur (rond a.u.b. af op hele dagen)

d. Weet ik niet

Q14. Is er een arts bezocht voor de tekenbeet?

a. Ja

b. Nee ga naar vraag: Q16

Q15. Welke arts is er bezocht? (meerdere antwoorden mogelijk)

a. Huisarts

b. Bedrijfsarts

c. Ander soort arts, namelijk: ..............

Q16. Is er een erythema migrans (rode ring) verschenen op de plek van de tekenbeet die u nu hebt gemeld?

a. Ja, de datum dat de erythema migrans verscheen .........(dag/maand/jaar)

b. Nee ga naar: Q17

Indien Q16=A deelnemer wordt EM-melder

Q17. Heeft een arts bij u ooit eerder een 'erythema migrans' of een andere uiting van 'de ziekte van Lyme' vastgesteld?

a. Nee ga naar vraag: Q21

b. Ja

Q18. Is er een antibiotica kuur verstrekt voor deze eerdere erythema migrans of andere uiting van 'de ziekte van Lyme'?

a. Nee

b. Ja; hoe vaak: .......Antibioticakuren

c. Onbekend

Q206. Bent u toen hersteld van de eerdere erythema migrans of andere uiting van de ziekte van Lyme?

a. Ja

b. Nee, ik ben klachten blijven houden tot op heden

Q21. Heeft u in de afgelopen 2 weken medicijnen gebruikt? Zo ja, welke medicijnen en tegen welke gezondheidsklachten?

a. Nee

b. Ja, namelijk (bijvoorbeeld antibiotica, paracetemol, etc):

Medicijn: ............. Tegen EM/ziekte van Lyme ///

Tegen iets anders dan de ziekte van Lyme

Indien geen deelnemer toevoegen:

Q?????. Mogen wij u over 3 maanden een email sturen om te vragen hoe het verder gegaan is?

1. Ja
2. Nee

Indien niet ingelogd hoofdstuk Registreren? toevoegen:

### Registreren?

**Ik heb geen account**

Met een account kunt u:

•Tekenbeten en erythema migrans(?) registreren voor uzelf en uw gezinsleden

•Inzicht krijgen in uw geregistreerde tekenbeten en erythema migrans(?)

•Bijdragen aan onderzoek naar tekenbeten en de ziekte van Lyme

U kunt eventueel bij een volgende registratie alsnog een account aanmaken.

Account aanmaken (klikbaar)

Doorgaan zonder account (klikbaar)

**Ik heb al een account**

E-mailadres: …….……………….

Wachtwoord: …….……………..

Inloggen (klikbaar)

Wachtwoord vergeten? (klikbaar)

Indien geen deelnemer -> Afsluiting 1

### Algemene gezondheid (TB-E1)

Wordt per jaar 1x getoond aan iedere deelnemer -> indien dit jaar al ingevuld: overslaan

Algemene gezondheid

Tekenbeet melden voor: mijzelf (voornaam achternaam)

Q28. U ziet hier een lijst met chronische aandoeningen en ziekten. Wilt u elke ziekte of aandoening aankruisen die u heeft, of die u in het afgelopen jaar heeft gehad? (meerdere antwoorden mogelijk)

1. Astma, chronische bronchitis of CARA
2. Ontsteking van de neusbijholte, voorhoofdsholte of kaakholten
3. Ernstige hartkwaal of hartinfarct
4. Hoge bloeddruk
5. Beroerte of gevolgen van beroerte
6. Maagzweer of zweer aan de 12-vingerige darm
7. Ernstige darmstoornissen, langer dan 3 maanden
8. Galstenen of galblaasontsteking
9. Leverziekte of levercirrose
10. Nierstenen
11. Ernstige nierziekte
12. Chronische blaasontsteking
13. Verzakking
14. Suikerziekte
15. Schildklierafwijking
16. Rugaandoening van hardnekkige aard, langer dan 3 maanden,of hernia
17. Gewrichtsslijtage (artrose) van knieën, heupen of handen
18. Gewrichtsontsteking (reuma) van handen en/of voeten
19. Andere chronische reuma, langer dan 3 maanden
20. Epilepsie
21. Andere ziekten van het zenuwstelsel, zoals ziekte van Parkinson
22. Multiple sclerose
23. Duizeligheid met vallen
24. Migraine
25. Kwaadaardige aandoening of kanker
26. Overspannen, depressie, ernstige nervositeit
27. Chronische huidziekte of eczeem
28. Letsel door ongeluk in en om huis sport, school, werk of in het verkeer
29. Afweerstoornis
30. Ondergaan van transplantatie
31. Alcoholverslaving
32. Drugsverslaving
33. Geen van de bovenstaande

Indien Q16=a ->als de gemelde TB al een EM veroorzaakt heeft, wordt persoon EM melder

Indien de deelnemer is aangemeld/ingelogd: - direct doorsturen naar: Uploaden foto (EM-E)

Indien de deelnemer niet is aangemeld/ingelogd:

Q??? U heeft aangegeven dat op de plek van de tekenbeet die u zojuist heeft gemeld ook een erythema migrans verschenen is. Bent u bereid om een foto van uw erythema migrans op te sturen en mee te doen met onderzoek van het RIVM naar tekenbeten en de ziekte van Lyme?

Ja ga naar Q52 ( Uploaden foto)

Nee ga naar Afsluiting 1???

### Algemeen welbevinden (korte versie) (TB-E2)

Als EM melder (start = EM | q16 = a) -> Q72 (Algemeen welbevinden (lange versie) TB-G2)

Algemeen welbevinden

Tekenbeet melden voor: mijzelf (margriet harms)

Q29. Wat vindt u, over het algemeen genomen, van uw gezondheid? (q29)

a. Uitstekend

b. Zeer goed

c. Goed

d. Matig

e. Slecht

De volgende vragen gaan over dagelijkse bezigheden. Wordt u door uw gezondheid op dit moment beperkt bij deze bezigheden? Zo ja, in welke mate?

|  | Ja, ernstig beperkt | Ja, een beetje beperkt | Nee, helemaal niet beperkt |
| --- | --- | --- | --- |
| Q30. Matige inspanning, zoals het verplaatsen van een tafel, stofzuigen, fietsen |  |  |  |
| Q31. Een paar trappen oplopen |  |  |  |

Had u, ten gevolge van uw lichamelijke gezondheid, de afgelopen 4 weken één van de volgende problemen bij uw werk of andere dagelijkse bezigheden?

|  | Ja | Nee |
| --- | --- | --- |
| Q32. U heeft minder bereikt dan u zou willen |  |  |
| Q33. U was beperkt in het soort werk of het soort bezigheden |  |  |

Had u, tengevolge van een emotioneel probleem (bijvoorbeeld doordat u zich depressief of angstig voelde), de afgelopen 4 weken één van de volgende problemen bij uw werk of andere dagelijkse bezigheden?

|  | Ja | Nee |
| --- | --- | --- |
| Q34. U heeft minder bereikt dan u zou willen |  |  |
| Q35. U heeft werk of andere bezigheden niet zo zorgvuldig gedaan als u gewend bent |  |  |

Q36. In welke mate heeft pijn u de afgelopen vier weken belemmerd bij uw normale werkzaamheden (zowel werk buitenshuis als huishoudelijk werk)?

a. Helemaal niet

b. Enigzins

c. Nogal

d. Veel

e. Heel erg veel

Deze vragen gaan over hoe u zich de afgelopen 4 weken heeft gevoeld. Wilt u bij elke vraag het antwoord aankruisen dat het beste aansluit bij hoe u zich heeft gevoeld?

Hoe vaak gedurende de afgelopen 4 weken:

|  | Voortdurend | Meestal | Vaak | Soms | Zelden | Nooit |
| --- | --- | --- | --- | --- | --- | --- |
| Q37. Voelde u zich kalm en rustig? |  |  |  |  |  |  |
| Q38. Voelde u zich erg energiek? |  |  |  |  |  |  |
| Q39. Voelde u zich neerslachtig en somber? |  |  |  |  |  |  |

Q40. Hoe vaak hebben uw lichamelijke gezondheid of emotionele problemen gedurende de afgelopen 4 weken uw sociale activiteiten (zoals bezoek aan vrienden of naaste familieleden) belemmerd? (q40)

a. Voortdurend

b. Meestal

c. Soms

d. Zelden

e. Nooit

### Verwachtingen klachtenbeloop (TB-E3)

Verwachtingen klachtenbeloop

Tekenbeet melden voor: mijzelf (margriet harms)

Hieronder vindt u een aantal uitspraken over uw verwachtingen ten aanzien van de tekenbeet die u hebt opgelopen. Geef van elke uitspraak aan in hoeverre deze op u van toepassing is.

|  | Helemaal niet mee eens | Niet mee eens | Niet eens/ niet oneens | Mee eens | Helemaal mee eens |
| --- | --- | --- | --- | --- | --- |
| Q41. Ik maak me zorgen over de mogelijke gevolgen van mijn tekenbeet |  |  |  |  |  |
| Q42. Ik verwacht dat mijn tekenbeet ernstige gevolgen voor mijn gezondheid zal hebben. |  |  |  |  |  |
| Q43. Ik verwacht dat ik nog lang last zal hebben van de gevolgen van mijn tekenbeet. |  |  |  |  |  |
| Q44. Ik verwacht dat ik in de toekomst geen medische hulp meer behoef voor de gevolgen van mijn tekenbeet. |  |  |  |  |  |
| Q45. Ik verwacht dat ik me in de toekomst geen zorgen meer hoef te maken over de gevolgen van mijn tekenbeet. |  |  |  |  |  |
| Q46. Ik verwacht dat mijn (eventuele) klachten ten gevolge van de tekenbeet geheel zullen verdwijnen |  |  |  |  |  |

### Teek opsturen (TB-B2)

Geef hieronder aan of u een grote of kleine teek gaat opsturen.

Tekenbeet melden voor: mijzelf (voornaam achternaam)

Q24. Selecteer: (Zie Fig. 2)

Dik. Grote teek

Is de teek of is één van de teken een grote volgezogen teek, zoals de 2 teken op de onderstaande foto (zie ook de afmetingen in centimeters)

Dun. Kleine teek

Is de teek of zijn al de teken kleine, niet volgezogen teken zoals de teken op de afbeelding hieronder


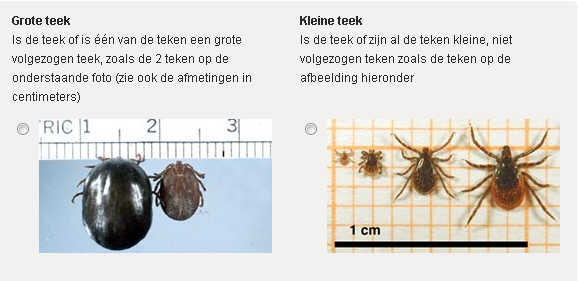


Fig. 2: Vraag 47 (Deelnemers kunnen aangeven of zij een kleine of grote teek op willen sturen door te klikken op de afbeeldingen)

Indien de deelnemer aangeeft een grote teek op te willen sturen:

#### Grote teek opsturen

Tekenbeet melden voor: mijzelf ( voornaam achternaam)

Voor het versturen van uw grote volgezogen teek sturen wij u binnen 3 werkdagen een speciaal verzendpakket toe. Dit verzendpakket bestaat uit een envelop met daarin een onderzoeksbuisje waarin de teek zonder risico op beschadiging naar het RIVM gestuurd kan worden. In het pakket zitten ook de verdere instructies voor verzending. Als u meer dan één teek heeft dan kunt u deze gezamenlijk opsturen in hetzelfde buisje.

Het is belangrijk om de teek/teken veilig te bewaren totdat u ze opstuurt. Vaak leven teken namelijk nog nadat ze van de huid verwijderd zijn. Stop de teek/teken daarom in een afgesloten bakje zoals een lenshouder of een doosje van een fotorolletje. Eventueel kunt u de teek/teken ook in een dichtgeknoopt plastic zakje in een gewoon doosje bewaren. Het is belangrijk dat er geen gaatjes zijn waardoor de teek kan ontsnappen EN dat de teek voldoende beschermd is zodat hij niet platgedrukt kan worden.

U mag uw ondertekende toestemmingsverklaring samen met de teek opsturen, gebruikt u wel de bubbel-envelop uit het pakketje hiervoor.

Indien de deelnemer aangeeft een kleine teek op te willen sturen:

#### Kleine teek opsturen

Tekenbeet melden voor: mijzelf (margriet harms)

Kleine teken kunt u met een plakbandje in het daarvoor bestemde vakje op de toestemmingsverklaring plakken (zie voorbeeld hieronder). Eventueel kunt u hier ook een stuk karton of en ander papier voor gebruiken.


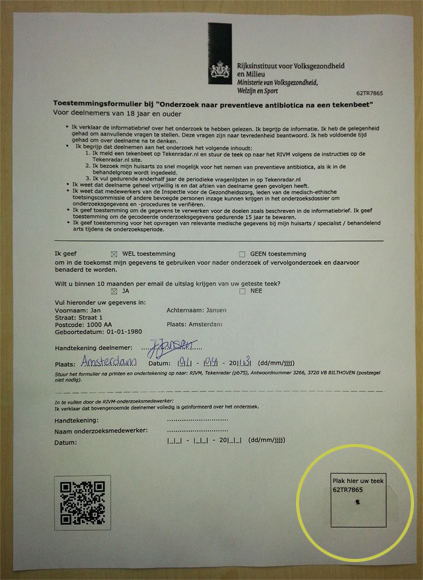


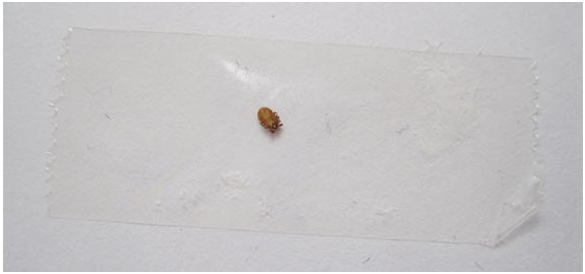


Meerdere kleine/niet volgezogen teken van dezelfde persoon, kunnen gezamenlijk op één vel papier verzonden worden.

Vermeld op het vel papier bij de teek de naam van de persoon die gebeten is door deze teek, de adresgegevens en het onderstaande identificatienummer. (In de toestemmingsverklaring staan deze reeds vooringevuld)

identificatienummer: xxxxTRxxxx

(Dit is het IDnummer waar de teek aan herkent kan worden op het moment dat hij per post bij ons binnen komt. Dit zelfde nummer zit gekoppeld aan de datasets)


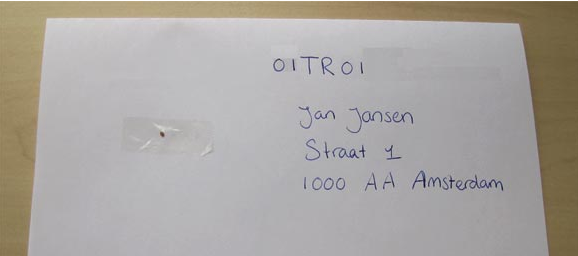


Fig. 4: Voorbeeld hoe de teek naar het RIVM dient opgestuurd te worden

U kunt de opgeplakte teek met bijgeschreven informatie aan ons versturen in een gesloten envelop naar onderstaand antwoordnummer (geen postzegel nodig):

RIVM

Tekenradar (pb 75)

Antwoordnummer 3266

3720 VB BILTHOVEN

De teek mag in dezelfde enveloppe als uw ondertekende toestemmingsverkaring verstuurd worden. Op de toestemmingsverklaring zelf is eventueel ook ruimte gereserveerd waar u de teek kunt plakken.

Indien deelnemer -> Afsluiting 2 (startvragenlijst TB deelname)

### Afsluiting 1 (anonieme melding)

Hartelijk dank voor het invullen van deze vragenlijst

Indien Q16 = a

U heeft aangegeven dat de tekenbeet een EM veroorzaakt heeft, klik hier om deze EM te melden een foto te uploaden -> ga naar: …………….

Het is belangrijk dat u de komende maanden de plek van de tekenbeet goed in de gaten houdt. Ga naar de huisarts als een erythema migrans(?) ontstaat of als u klachten krijgt die op de ziekte van Lyme kunnen duiden. Hier vindt u meer informatie over in welke situaties het verstandig is de huisarts te bezoeken.

Hartelijk bedankt voor uw deelname aan ons onderzoek!

Indien q????=a (mogen we u reminden over 3 maanden????)

Over 3 maanden ontvangt u van ons een e-mail met een uitnodiging voor een vragenlijst waarin wij vragen of er bij u klachten zijn ontstaan sinds de tekenbeet.

Heeft u nog een opmerking of extra informatie over de door u ingevulde vragenlijst?

...................................................

(Let op: deze informatie wordt bij de door u ingevulde vragenlijst opgeslagen, heeft u een vraag dan kunt u deze hier stellen)

### Afsluiting 2 (startvragenlijst TB deelname)

Hartelijk dank voor het invullen van deze vragenlijst

Indien deelnemer een nieuwe account heeft aangemaakt:

Er is een email naar u verstuurd waarin staat hoe u uw deelname aan Tekenradar.nl kunt bevestigen. In een tweede email vindt u nogmaals de patientinformatie van dit onderzoek en uw toestemmingsverklaring.

Indien de deelnemer reeds een bestaande account heeft en hierop heeft ingelogd:

Er is een email naar u verstuurd waarin u nogmaals de patientinformatie van dit onderzoek vindt en uw toestemmingsverklaring.

Heeft u de toestemmingsverklaring nog niet uitgeprint en ondertekend, of de teek nog niet opgestuurd, zou u dit dan zo snel mogelijk willen doen. Instructies voor het opsturen van de teek vindt u hier.

Indien de patient is ingedeeld in de behandelgroep deze alinea toevoegen:

Bij de email zit ook een bijlage met informatie voor uw huisarts. Print deze uit en neem deze mee als u de preventieve antibiotica gaat halen.

Volgende week krijgt u van ons een e-mail toegestuurd met een link naar een korte vragenlijst.

Indien q16=b (geen EM ontstaan)

Het is belangrijk dat u de komende maanden de plek van de tekenbeet goed in de gaten houdt. Ga naar de huisarts als een erythema migrans ontstaat of als u klachten krijgt die op de ziekte van Lyme kunnen duiden.

Hier vindt u meer informatie over in welke situaties het verstandig is de huisarts te bezoeken.

Indien Q16=a (wel EM onstaan)

Als u nog niet bij de huisarts bent geweest voor deze erythema migrans, dan adviseren wij u zo snel mogelijk contact op te nemen met uw huisarts voor behandeling van uw erythema migrans(?).

Hartelijk bedankt voor uw deelname aan ons onderzoek!

Heeft u nog een opmerking of extra informatie over de door u ingevulde vragenlijst?

...................................................

(Let op: deze informatie wordt bij de door u ingevulde vragenlijst opgeslagen, vragen over het onderzoek kunt u hier stellen)

## Erythema migrans melding

Start: Tekenbeet melden -> zie routing tekenbeet melding (Melden tekenbeet (TB-A))

Erythema migrans melden

### Registreren?

**Ik heb geen account**

Met een account kunt u:

•Tekenbeten en erythema migrans(?) registreren voor uzelf en uw gezinsleden

•Inzicht krijgen in uw geregistreerde tekenbeten en erythema migrans(?)

•Bijdragen aan onderzoek naar tekenbeten en de ziekte van Lyme

U kunt eventueel bij een volgende registratie alsnog een account aanmaken.

Account aanmaken (klikbaar) -> account aanmaken

**Ik heb al een account**

E-mailadres:………….

Wachtwoord:……………….

Inloggen (klikbaar)-> voor wie doet u deze melding

Wachtwoord vergeten? (klikbaar)> vul hier uw email in -> email verstuurd

### Account aanmaken

Account aanmaken

Voornaam:…………………………

Tussenvoegsel:…………………

Achternaam:……………………..

E-mailadres:…………………

Wachtwoord:……………..

Bevestig wachtwoord:……………….

Account aanmaken / Inloggen

### **Voor wie doet u deze melding?**

De onderstaande personen zijn aan uw account gekoppeld. Kies de persoon voor wie u deze melding doet.

Q1. Kies een persoon:

a. Mijzelf (voornaam achternaam)

b. Anders:

Voornaam: .............

Tussenvoegsel: ............

Achternaam: ................

### StartMelding (EM-A)

Erythema migrans(?) melden voor: mijzelf (voornaam achternaam)

U wilt een erythema migrans melden op Tekenradar.nl. Door uw melding neemt u deel aan onderzoek van het RIVM naar tekenbeten en de ziekte van Lyme. Voor dit onderzoek vragen wij u een vragenlijst in te vullen en een digitale foto te uploaden.

De komende anderhalf jaar sturen wij u elke 3 maanden een uitnodiging om via Tekenradar.nl een korte vragenlijst in te vullen over uw gezondheid. Eventueel stellen wij u nog aanvullende vragen over tekenbeten en de ziekte van Lyme. Als u opnieuw een teek op de huid vindt of als er een nieuwe erythema migrans op de huid ontstaat vragen wij u dat ook te melden op Tekenradar.nl. Uw gegevens worden vertrouwelijk en beveiligd bewaard en alleen gebruikt voor onderzoek naar tekenbeten en de ziekte van Lyme.

Q56. Heeft u de tekenbeet, waardoor u vermoedelijk de erythema migrans die u nu meldt heeft gekregen, opgemerkt?

a. Nee

b. Ja, deze heb ik eerder gemeld op Tekenradar.nl (pop-up, Fig. 2)

c. Ja, datum dat u de tekenbeet heeft opgelopen ..........(dag/maand/jaar) bij benadering?

d. Onbekend

Fig. 2: Pop-up waarin eerder geregistreerde tekenbeten worden weergegeven

### Melding EM (EM-B)

Erythema migrans(?) melden voor: mijzelf (margriet harms)

Q54. Wanneer ontwikkelde zich de huidige erythema migrans(?) die u nu meldt?

a. Datum dat de erythema migrans(?) zich ontwikkelde: .......... (dag/maand/jaar) bij benadering?

b.Onbekend

De volgende vragen gaan over de tekenbeet die mogelijk de huidige erythema migrans veroorzaakt heeft.

Q2. Is deze erythema migrans mogelijk veroorzaakt door een tekenbeet die u tijdens het werk heeft opgelopen?

a. Ja, mijn beroep is: ……..

b. Nee

c. Weet niet

Indien Q56 ≠ c -> ga naar: hoofdstuk: Achtergrond

(voor mensen die EM melden en Tbopgemerkt hebben, Q56=c )

Vragen Q3 t/m Q15 zijn identiek aan de vragen met hetzelfde nr. in TB-A, behalve toevoeging=….die de EM veroorzaakt heeft…

Q3. In welk type omgeving heeft u de tekenbeet die de erythema migrans veroorzaakt heeft opgelopen? (meerdere antwoorden mogelijk)

Q4. Bij welke activiteit heeft u de tekenbeet die de erythema migrans veroorzaakt heeft opgelopen? (meerdere antwoorden mogelijk)

Q7. Weet u de locatie waar u de tekenbeet die de erythema migrans veroorzaakt heeft (vermoedelijk) heeft opgelopen?

Q12. Wie heeft de teek verwijderd? (meerdere antwoorden mogelijk)

Q205. Wanneer heeft u de tekenbeet (vermoedelijk) opgelopen?

Q13. Hoe lang heeft de teek in de huid vastgezeten?

Q14. Is er een arts bezocht voor de tekenbeet?

Q15. Welke arts is er bezocht? (meerdere antwoorden mogelijk)

### Achtergrond (EM-C)

Wordt voor iedere deelnemer slechts 1x gevraagd

Erythema migrans melden voor: mijzelf (voornaam achternaam)

De volgende vragen gebruiken wij alleen om te bekijken of de deelnemers aan het onderzoek een goede afspiegeling zijn van de Nederlandse samenleving.

Q5. Wat is uw geboortejaar? …………..

Q????. Wat is uw geslacht?

1. Man
2. Vrouw

Q27. Wat is uw hoogst voltooide opleiding?

a. Geen voltooide opleiding (lager onderwijs niet afgemaakt)

b. Lager onderwijs (basisschool, speciaal basisonderwijs)

c. Lager of voorbereidend beroepsonderwijs (zoals LTS, LEAO, LHNO, VMBO)

d. Middelbaar algemeen voortgezet onderwijs (zoals MAVO, (M)ULO, MBO-kort, VMBO-t)

e. Middelbaar beroepsonderwijs en beroepsbegeleidend onderwijs (zoals MBO-lang, MTS, MEAO, BOL, BBL, INAS)

f. Hoger algemeen en voorbereidend wetenschappelijk onderwijs (zoals HAVO, VWO, Atheneum, Gymnasium, HBS,MMS)

g. Hoger beroepsonderwijs (zoals HBO, HTS, HEAO, kandidaatswetenschappelijk onderwijs)

h. Wetenschappelijk onderwijs (universiteit)

### Algemene gezondheid (EM-D)

Zie: Algemene gezondheid ( TB-E1)

### Uploaden foto (EM-E)

Tekenbeet melden voor: mijzelf (margriet harms)

Wij vragen u om een foto van uw erythema migrans. Mocht u nu geen foto kunnen uploaden, dan ontvangt u een reminder per email om dat later alsnog te doen.

Om een goed beeld te krijgen van de grootte van de erythema migrans stellen wij het zeer op prijs als er een lineaal of meetlint of een voorwerp met een standaard grootte zoals bijvoorbeeld een muntstuk naast de erythema migrans op de foto staat.

Q52. Selecteer hier de foto van uw erythema migrans

Indien start melding =TB +deelname (&q16=a) -> door naar Algemeen welbevinden (lange versie) EM-G2

Q51. Wat is uw adres?

Street. Adres (straat + huisnummer)

Postalcode. Postcode

City. Woonplaats

### Tekenbeten algemeen (EM-F)

Erythema migrans melden voor: mijzelf (test test test)

De volgende vragen gaan over de behandeling van uw erythema migrans en uw persoonlijke geschiedenis op het gebied van tekenbeten, erythema migrans en de ziekte van Lyme.

Q57. Bent u bij een arts geweest voor uw erythema migrans?

a. ja

b. nee > ga naar: Q61

Q58. Bij welke arts bent u toen geweest? (meerdere antwoorden mogelijk)

a. huisarts

b. bedrijfsarts

c. specialist

d. ander soort arts, namelijk:

Q59. Herkende de arts toen uw rode ring op de huid als een erythema migrans die waarschijnlijk is veroorzaakt door een tekenbeet?

a. ja

b. nee

Indien deelnemer TTPP deel, vraag Q254 toevoegen:

Q254. Wat zijn de gegevens van uw huisarts?

Naam huisarts: ………………

Plaats: …………………………..

Q60. Heeft u antibiotica gekregen voor uw erythema migrans?

a. Ja

b. nee >ga naar: Q61

Q138. Wanneer begon u met de antibiotica behandeling voor de ziekte van Lyme (inclusief erythema migrans) in de afgelopen 3 maanden?

Q252: Datum start kuur:………….., dat was:

a. na de tekenbeet (nog voordat u ziek werd en/of voordat een erythema migrans verscheen)

b. na het verschijnen van een erythema migrans

c. na het ontstaan van andere gezondheidsklachten door de ziekte van Lyme (anders dan erythema migrans)

d. weet ik niet

Q253 Hoeveel tijd zat er tussen de eerste gezondheidsklachten (inclusief erythema migrans) en het begin van de antibiotica kuur?

a. Minder dan 1 week

b. 1-2 weken

c. 2-4 weken

d. 1-2 maanden

e. 2-3 maanden

f. Langer, namelijk ….. maanden/jaren

Indien Q56=b: ga naar Algemeen welbevinden (lange versie) EM-G2

Q61. Heeft de huisarts bij u ooit eerder een ‘erythema migrans’ of een andere uiting van ‘de ziekte van Lyme’ vast gesteld?

a. nee >ga naar: Q63

b. ja

Q62. Heeft u een antibiotica kuur gekregen voor deze eerdere erythema migrans of andere uiting van ‘de ziekte van Lyme’?

a. nee

b. ja; hoe vaak: …. Antibioticakuren

c. onbekend

Q255. Bent u toen hersteld van de eerdere erythema migrans of andere uiting van de ziekte van Lyme?

a. Ja

b. Nee, ik ben klachten blijven houden tot op heden

Q63. Hoeveel tekenbeten heeft u **in de afgelopen 5 jaar** opgemerkt? (omdat u de tekenbeet die de huidige erythema migrans heeft veroorzaakt, niet heeft opgemerkt deze ook niet meetellen) [Hoeveel tekenbeten heeft u **in de afgelopen 5 jaar** opgemerkt als u de tekenbeet die de huidige erythema migrans heeft veroorzaakt **niet** meetelt?]

a. geen tekenbeten >Ga naar: Einde hoofdstuk

b. 1 - 3 tekenbeten

c. 4 - 10 tekenbeten

d. 11 - 50 tekenbeten

e. meer dan 50 tekenbeten

[vraagstelling in deze vraag wordt bepaald door antwoord op vraag Q56]

Q64. Hoeveel tekenbeten heeft u **in de afgelopen 3 maanden** opgemerkt? (omdat u de tekenbeet die de huidige erythema migrans heeft veroorzaakt, niet heeft opgemerkt deze ook niet meetellen) [Hoeveel tekenbeten heeft u **in de afgelopen 3 maanden** opgemerkt als u de tekenbeet die de huidige erythema migrans heeft veroorzaakt **niet** meetelt?]

a. geen tekenbeten

b. 1 - 3 tekenbeten

c. 4 - 10 tekenbeten

d. 11 - 50 tekenbeten

e. meer dan 50 tekenbeten

[vraagstelling in deze vraag wordt bepaald door antwoord op vraag Q56]

### Algemeen welbevinden (lange versie) EM-G2

Erythema migrans melden voor: mijzelf (voornaam achternaam)

De volgende vragen gaan over uw gezondheidstoestand in het algemeen.

Q72. Wat vindt u, over het algemeen genomen, van uw gezondheid? (q72)

a. Uitstekend

b. Zeer goed

c. Goed

d. Matig

e. Slecht

Q73. In vergelijking met een jaar geleden, hoe zou u nu uw gezondheid in het algemeen beoordelen?

a. Veel beter dan een jaar geleden

b. Iets beter dan een jaar geleden

c. Ongeveer hetzelfde als een jaar geleden

d. Iets slechter dan een jaar geleden

e. Veel slechter dan een jaar geleden

Q74. De volgende vragen gaan over dagelijkse bezigheden. Wordt u door uw gezondheid op dit moment beperkt bij deze bezigheden? Zo ja, in welke mate? (q74)

|  | Ja, ernstig beperkt | Ja, een beetje beperkt | Nee, helemaal niet beperkt |
| --- | --- | --- | --- |
| a. Forse inspanning; zoals hardlopen, zware voorwerpen tillen, inspannend sporten |  |  |  |
| b. Matige inspanning; zoals het verplaatsen van een tafel, stofzuigen, fietsen |  |  |  |
| c. Tillen of boodschappen dragen |  |  |  |
| d. Een paar trappen oplopen |  |  |  |
| e. Één trap oplopen |  |  |  |
| f. Buigen, knielen of bukken |  |  |  |
| g. Meer dan een kilometer lopen |  |  |  |
| h. Een halve kilometer lopen |  |  |  |
| i. Honderd meter lopen |  |  |  |
| j. Uzelf wassen of aankleden |  |  |  |

Q75. Had u, ten gevolge van uw lichamelijke gezondheid, de afgelopen 4 weken één van de volgende problemen bij uw werk of andere dagelijkse bezigheden? (q75)

|  | Ja | Nee |
| --- | --- | --- |
| a. U heeft minder tijd kunnen besteden aan werk of andere bezigheden |  |  |
| b. U heeft minder bereikt dan u zou willen |  |  |
| c. U was beperkt in het soort werk of het soort bezigheden |  |  |
| d. U had moeite met het werk of andere bezigheden (het kostte u bijvoorbeeld extra inspanning) |  |  |

Q76. Had u, tengevolge van een emotioneel probleem (bijvoorbeeld doordat u zich depressief of angstig voelde), de afgelopen 4 weken één van de volgende problemen bij uw werk of andere dagelijkse bezigheden? (q76)

|  | Ja | Nee |
| --- | --- | --- |
| a. U heeft minder tijd kunnen besteden aan werk of andere bezigheden |  |  |
| b. U heeft minder bereikt dan u zou willen |  |  |
| c. U heeft werk of andere bezigheden niet zo zorgvuldig gedaan als u gewend bent |  |  |

Q77. In hoeverre heeft uw lichamelijke gezondheid of hebben uw emotionele problemen u de afgelopen 4 weken belemmerd in uw normale sociale bezigheden met gezin, vrienden, buren of anderen? (q77)

a. Helemaal niet

b. Enigzins

c. Nogal

d. Veel

e. Heel erg veel

Q78. Hoeveel pijn had u de afgelopen 4 weken? (q78)

a. Geen

b. Heel licht

c. Licht

d. Nogal

e. Ernstig

f. Heel ernstig

Q79. In welke mate heeft pijn u de afgelopen vier weken belemmerd bij uw normale werkzaamheden (zowel werk buitenshuis als huishoudelijk werk)? (q79)

a. Helemaal niet

b. Enigzins

c. Nogal

d. Veel

e. Heel erg veel

Q80. Deze vragen gaan over hoe u zich de afgelopen 4 weken heeft gevoeld. Wilt u bij elke vraag het antwoord aankruisen dat het beste aansluit bij hoe u zich heeft gevoeld? Hoe vaak gedurende de afgelopen 4 weken: (q80)

|  | Voordurend | Meestal | Vaak | Soms | Zelden | Nooit |
| --- | --- | --- | --- | --- | --- | --- |
| a. Voelde u zich levenslustig |  |  |  |  |  |  |
| b. Voelde u zich erg zenuwachtig |  |  |  |  |  |  |
| c. Zat u zo erg in de put dat niets u kon opvrolijken? |  |  |  |  |  |  |
| d. Voelde u zich kalm en rustig? |  |  |  |  |  |  |
| e. Voelde u zich erg energiek? |  |  |  |  |  |  |
| f. Voelde u zich neerslachtig en somber? |  |  |  |  |  |  |
| g. Voelde u zich uitgeblust? |  |  |  |  |  |  |
| h. Voelde u zich gelukkig? |  |  |  |  |  |  |
| i. Voelde u zich moe? |  |  |  |  |  |  |

Q81. Hoe vaak hebben uw lichamelijke gezondheid of emotionele problemen gedurende de afgelopen 4 weken uw sociale activiteiten (zoals bezoek aan vrienden of naaste familieleden) belemmerd? (q81)

a. Voortdurend

b. Meestal

c. Soms

d. Zelden

e. Nooit

Q82. Wilt u het antwoord kiezen dat het beste weergeeft hoe juist of onjuist u elk van de volgende uitspraken voor uzelf vindt? (q82)

|  | Volkomen juist | Grotendeels juist | Weet ik niet | Grotendeels onjuist | Volkomen onjuist |
| --- | --- | --- | --- | --- | --- |
| a. Ik lijk gemakkelijker ziek te worden dan andere mensen |  |  |  |  |  |
| b. Ik ben net zo gezond als andere mensen die ik ken |  |  |  |  |  |
| c. Ik verwacht dat mijn gezondheid achteruit zal gaan |  |  |  |  |  |
| d. Mijn gezondheid is uitstekend |  |  |  |  |  |

### Verwachtingen klachtenbeloop (EM-G3)

Erythema migrans melden voor: mijzelf (voornaam achternaam)

Hieronder vindt u een aantal uitspraken over uw verwachtingen ten aanzien van de erythema migrans(?) die u heeft opgelopen. Geef van elke uitspraak aan in hoeverre deze op u van toepassing is.

|  | Helemaal niet mee eens | Niet mee eens | Niet eens/ niet oneens | Mee eens | Helemaal mee eens |
| --- | --- | --- | --- | --- | --- |
| Q83. Ik maak me zorgen over de mogelijke gevolgen van de erythema migrans |  |  |  |  |  |
| Q84. Ik verwacht dat de erythema migrans ernstige gevolgen voor mijn gezondheid zal hebben |  |  |  |  |  |
| Q85. Ik verwacht dat ik nog lang last zal hebben van de gevolgen van de erythema migrans |  |  |  |  |  |
| Q86. Ik verwacht dat ik in de toekomst geen medische hulp meer behoef voor de gevolgen van de erythema migrans |  |  |  |  |  |
| Q87. Ik verwacht dat ik me in de toekomst geen zorgen meer hoef te maken over de gevolgen van de erythema migrans |  |  |  |  |  |
| Q88. Ik verwacht dat mijn (eventuele) klachten ten gevolge van de erythema migrans(?) geheel zullen verdwijnen |  |  |  |  |  |

### Klachtenperceptie (EM-G4)

Klachtenperceptie

Erythema migrans melden voor: mijzelf (voornaam achternaam)

De volgende vragen gaan over uw gezondheidsklachten door de erythema migrans(?).

Q89. Hoeveel beïnvloeden uw klachten ten gevolge van de erythema migrans(?) uw leven?

0 1 2 3 4 5 6 7 8 9 10

Helemaal geen invloed Zeer veel invloed

Q90. Hoe lang denkt u dat de klachten ten gevolge van de erythema migrans(?) zullen duren?

0 1 2 3 4 5 6 7 8 9 10

Een zeer korte tijd Mijn hele leven

Q91. Hoeveel controle vindt u dat u heeft over uw klachten ten gevolge van de erythema migrans(?)?

0 1 2 3 4 5 6 7 8 9 10

Helemaal geen controle Zeer veel controle

Q92. Hoeveel denkt u dat een behandeling kan helpen bij uw klachten ten gevolge van de erythema migrans(?)?

0 1 2 3 4 5 6 7 8 9 10

Helemaal niet Zeer veel

Q93. Hoe sterk ervaart u klachten door de erythema migrans(?)?

0 1 2 3 4 5 6 7 8 9 10

Helemaal geen klachten Veel ernstige klachten

Q94. Hoe bezorgd bent u over uw klachten ten gevolge van de erythema migrans(?)?

0 1 2 3 4 5 6 7 8 9 10

Helemaal niet bezorgd Zeer bezorgd

Q95. In welke mate vindt u dat u uw klachten ten gevolge van de erythema migrans(?) begrijpt?

0 1 2 3 4 5 6 7 8 9 10

Helemaal geen begrip Zeer veel begrip

Q96. Hoeveel invloed hebben uw klachten ten gevolge van de erythema migrans(?) op uw stemming?

0 1 2 3 4 5 6 7 8 9 10

Helemaal geen invloed Zeer veel invloed

### Zorgconsumptie (EM-G5)

Erythema migrans melden voor: mijzelf (voornaam achternaam)

Dit deel van de vragenlijst is bedoeld om in kaart te brengen met welke zorg- of hulpverleners u voor uzelf in de afgelopen 3 maanden contact hebt gehad vanwege de erythema migrans(?).

Q97. Heeft u in de afgelopen 3 maanden vanwege de erythema migrans(?) contact gehad met één of meerdere zorg- of hulpverleners (bijvoorbeeld huisarts, bedrijfsarts, ziekenhuis, fysiotherapeut, alternatieve genezer, maatschappelijk werker of andere hulpverlener)?

a. Ja

b. Nee ga naar: Einde van dit hoofdstuk

Tel voor het aantal contacten alle spreekuren, bezoeken op afspraak, telefonische contacten en huisbezoeken mee. Telefonische contacten met de secretaresse of assistenten van een hulpverlener om een afspraak te maken dienen niet meegeteld te worden.

Als u een antwoord niet precies weet, mag u gerust een schatting geven.

Q98. Hebt u in de afgelopen 3 maanden contact gehad met de huisarts vanwege de erythema migrans(?)?

a. Nee

b. Ja, namelijk ...... contact(en)

(Wilt u alle spreekuurbezoek, bezoek op afspraak, huisbezoek en telefonische consulten vanwege de erythema migrans(?) in de afgelopen 3 maanden bij elkaar optellen.)

Q99. Hebt u in de afgelopen 3 maanden contact gehad met een bedrijfsarts vanwege de erythema migrans(?)?

a. Nee

b. Ja, namelijk ...... contact(en)

Q100. Hebt u in de afgelopen 3 maanden vanwege de erythema migrans(?) contact gehad met een medisch specialist op de polikliniek van een ziekenhuis zonder dat u was opgenomen in het ziekenhuis?

(Voorbeelden van medische specialisten zijn: cardioloog, reumatoloog, internist of neuroloog.)

a. Nee

b. Ja, namelijk

Specialist: ...................

Aantal contacten in de afgelopen 3 maanden: ...........

Q101. Hebt u in de afgelopen 3 maanden contact gehad met een paramedicus vanwege de erythema migrans(?)? (Voorbeelden van een paramedicus zijn: fysiotherapeut, logopedist, Caesartherapeut, ergotherapeut, manueel therapeut, therapeut Mensendieck)

a. Nee

b. Ja, namelijk ........ contact(en)

Q102. Hebt u in de afgelopen 3 maanden contact gehad met een maatschappelijk werk(st)er vanwege de erythema migrans(?)?

a. Nee

b. Ja, namelijk ............ contact(en)

Q103. Hebt u in de afgelopen 3 maanden contact gehad met een alternatieve genezer vanwege de erythema migrans(?)? (Voorbeelden van een alternatieve genezer zijn: homeopaat, acupuncturist, natuurgenezer, iriscopist, holistisch therapeut, paragnost, Reikitherapeut.)

a. Nee

b. Ja, namelijk ............ contact(en)

Q104. Hebt u in de afgelopen 3 maanden een dagopname in een instelling (bv. een ziekenhuis) vanwege de erythema migrans(?) gehad? Een dagopname kan variëren van een dagdeel tot 5 dagen per week, maar zonder in de instelling te overnachten en niet op de poli-kliniek. (Wilt u alle dagen en dagdelen bij elkaar optellen die u de afgelopen 3 maanden hebt doorgebracht in dagopname?)

a. Nee ga naar vraag Q106

b. Ja, namelijk ........ dagen

Q105 In welk soort instelling was dit?

a. Academisch ziekenhuis

b. Algemeen ziekenhuis

c. Een ander soort instelling, namelijk: ............................

Q106. Bent u in de afgelopen 3 maanden opgenomen geweest in een instelling binnen de gezondheidszorg vanwege de erythema migrans(?)? (d.w.z. één nacht of meer in bijvoorbeeld ziekenhuis)

a. Nee ga naar vraag Q108

b. Ja, namelijk ....... dagen

Q107. In welk soort instelling was dit?

a. Academisch ziekenhuis

b. Algemeen ziekenhuis

c. Een ander soort instelling, namelijk: .........................

Q108. Hoe bent u tegen ziektekosten verzekerd?

a. Basispakket

b. Basis+ aanvullend pakket

c. Niet verzekerd

### Ziekteverzuim (EM-G6)

Tekenbeet melden voor: mijzelf (margriet harms)

De volgende vragen gaan over de gevolgen van erythema migrans(?) voor betaald werk en onbetaald werk (bijvoorbeeld bij huishoudelijk werk). Deze vragen hebben steeds betrekking op de **afgelopen maand**. Met gezondheidsproblemen worden zowel uw lichamelijke als emotionele problemen bedoeld.

Q109. Hebt u momenteel betaald werk?

a. Nee ga naa vraag Q118

b. Ja

Voor hoeveel uur per week hebt u een aanstelling? .......... uren per week

Over hoeveel dagen zijn deze uren verdeeld? .......... dagen

Wat is uw beroep? .....................

Q110. Hebt u de afgelopen maand verzuimd van betaald werk vanwege de erythema migrans(?)?

a. Nee

b. Ja, ik heb ............. werkdagen verzuimd (Ga uit van 5 werkdagen per week)

Q111. Hebt u langer dan de gehele afgelopen maand verzuimd van betaald werk vanwege de erythema migrans(?)?

a. Nee

b. Ja, ik heb mij ziek gemeld sinds .......... (dag/maand/jaar) ga naar vraag Q117

Mensen met gezondheidsproblemen moeten daarvoor soms verzuimen van hun werk. Het kan echter voorkomen dat iemand wel op zijn werk aanwezig is, maar zijn werk minder goed doet vanwege die gezondheidsproblemen. Daarover gaan de volgende vragen.

Q112. Werd u in de afgelopen maand bij uw betaald werk gehinderd door de erythema migrans(?) ?

a. Nee, in het geheel niet ga naar vraag Q117

b. Ja, een beetje

c. Ja, heel erg

Q113. Hoeveel dagen in de afgelopen maand hebt u wèl betaald werk verricht, terwijl u last had van de erythema migrans?

.......... dagen (De dagen waarop u helemaal niet hebt gewerkt omdat u zich ziek hebt gemeld hoeft u niet mee te rekenen)?

Q114. Wilt u aangeven hoe goed u hebt gewerkt op de dagen dat u wèl op uw werk was terwijl u last had van de erythema migrans. (Een 1 betekent dat u zeer slecht in staat was uw werk uit te voeren en een 10 betekent dat uw werk niet werd beïnvloed.)

1 2 3 4 5 6 7 8 9 10

Zeer slecht Even goed als normaal

Nu volgt een aantal uitspraken die voor mensen met een erythema migrans/de ziekte van Lyme van toepassing kunnen zijn met betrekking tot betaald werk. Geef aan hoe vaak elke uitspraak op u van toepassing was in de afgelopen maand.

Q115. Ik was wel op mijn werk, maar als gevolg van de erythema migrans(?)

|  | Bijna nooit | Soms | Vaak | Bijna altijd |
| --- | --- | --- | --- | --- |
| a. had ik concentratiestoornissen |  |  |  |  |
| b. moest ik in een langzamer tempo werken |  |  |  |  |
| c. moest ik mij afzonderen |  |  |  |  |
| d. had ik meer problemen om beslissingen te nemen |  |  |  |  |
| e. moest ik werk uitstellen |  |  |  |  |
| f. moest ik werk laten overnemen door anderen |  |  |  |  |
| g. had ik andere problemen, nl |  |  |  |  |

Q116. Als u het werk zou moeten inhalen dat u in de afgelopen maand niet hebt kunnen verrichten vanwege de erythema migrans(?), hoeveel uur zou u dan moeten werken?

.......... uur (De dagen waarop u helemaal niet hebt gewerkt omdat u zich ziek hebt gemeld hoeft u niet mee te rekenen.)

Q117. Wat is uw eigen netto inkomen uit betaald werk? (Het gaat om het bedrag dat u ‘schoon’ in uw handen krijgt. Voor alle duidelijkheid: het gaat alleen om uw eigen inkomen, dus zonder dat van uw eventuele partner.)

a. ...........€ per week

b. ...........€ per 4 weken

c. ...........€ per maand

d. ...........€ per jaar

e. Weet ik niet / wil ik niet zeggen

Q118. Welke van de volgende situaties is op u van toepassing? Indien meerdere situaties van toepassing zijn, wilt u dan aangeven welke situatie het meest op u van toepassing is?

a. Ik heb een betaalde baan

b. Ik zorg voor het huishouden (en eventueel kinderen)

c. Ik ben gepensioneerd of met prepensioen

d. Ik ben scholier of student

e. Ik kan (gedeeltelijk) geen betaald werk doen vanwege gezondheidsproblemen en ben voor …..% arbeidsgeschikt

f. Ik doe geen betaald werk om andere redenen (Bijv. vanwege onvrijwillige werkloosheid of vrijwilligerswerk.)

Q119. Bent u in de afgelopen maand gehinderd bij activiteiten in uw vrije tijd door de erythema migrans?

a. Nee, in het geheel niet ga naar Einde van dit hoofdstuk

b. Ja, een beetje

c. Ja, heel erg

Hebt u de afgelopen maand de volgende activiteiten verricht en heeft de erythema migrans daarbij een rol gespeeld?

Q120. Huishoudelijk werk (bijv. eten klaar maken, huis schoonmaken, kleren wassen.)

a. Wel gedaan, niet gehinderd door de erythema migrans

b. Wel gedaan, wel gehinderd door de erythema migrans

c. Niet gedaan, vanwege de erythema migrans

d. Niet gedaan, om andere redenen dan de erythema migrans

Q121. Boodschappen doen (bijv. dagelijkse boodschappen, winkelen, bezoek aan bank of postkantoor.)

a. Wel gedaan, niet gehinderd door de erythema migrans

b. Wel gedaan, wel gehinderd door de erythema migrans

c. Niet gedaan, vanwege de erythema migrans

d. Niet gedaan, om andere redenen dan de erythema migrans

Q122. Klussen en karweitjes (bijv. onderhoud aan huis, tuin of vervoermiddelen.)

a. Wel gedaan, niet gehinderd door de erythema migrans

b. Wel gedaan, wel gehinderd door de erythema migrans

c. Niet gedaan, vanwege de erythema migrans

d. Niet gedaan, om andere redenen dan de erythema migrans

Q123. Dingen speciaal voor of met uw eigen inwonende kinderen (bijv. verzorging, spelen, kinderen naar school brengen, helpen met huiswerk.)

a. Wel gedaan, niet gehinderd door de erythema migrans

b. Wel gedaan, wel gehinderd door de erythema migrans

c. Niet gedaan, vanwege de erythema migrans

d. Niet gedaan, om andere redenen dan de erythema migrans

e. Niet van toepassing

Q124. Hebben anderen in de afgelopen maand huishoudelijke taken die u normaal wel doet overgenomen in verband met de erythema migrans?

a. Nee

b. Ja, namelijk (meerdere antwoorden mogelijk):

Gezinsleden voor ........... uur

Andere onbetaalde mensen voor ........... uur

Thuiszorg voor ........ uur

Andere betaalde hulp voor .......... uur

Indien TB+EM gemeld -> Teek opsturen (TB-B2)

### Afsluiting 3 (losse EM melding)

Hartelijk dank voor het invullen van deze vragenlijst

Over 3 maanden ontvangt u van ons een e-mail op het door u opgegeven e-mailadres met een link naar een vragenlijst waarin wij opnieuw informeren naar uw gezondheid.

Elke nieuwe tekenbeet of erythema migrans(?) mag u melden op Tekenradar.nl.

Heeft u nog een opmerking of extra informatie over de door u ingevulde vragenlijst?

...........................

........................

(Let op: deze informatie wordt bij de door u ingevulde vragenlijst opgeslagen, vragen over het onderzoek kunt u hier stellen)

## Vervolg vragenlijsten

T=1 week

### Vervolgvragenlijst T = 1 week na inclusie

Voor: onderzoeksdeelnemers met startpunt = TB onafhankelijk of al een EM gemeld is

Uitnodiging gaat via een e-mail met daarin een direct link naar de vragenlijst

Er zijn 2 versies voor: -niet-behandelgroep

-behandelgroep

Deze indeling is bepaald door de randomizer na accoord gaan met de toestemmingsverklaring

#### Antibiotica

Hieronder worden een aantal vragen gesteld over de afgelopen week.

Voor de onderzoeksgroep: niet-behandelen:

CG1. Heeft u sinds de vorige vragenlijst uw huisarts bezocht voor uw tekenbeet?

a. Nee Ga naar: Diagnoses

b. Ja, vul hier de reden in:……..……

CG2. Heeft uw huisarts antibiotica voorgeschreven?

a.Ja, omdat:……………..

b.Nee Ga naar: Diagnoses

CG3. Heeft u de antibiotica ingenomen?

a. Ja

b. Nee Ga naar: Diagnoses

CG4. Wanneer heeft u de antibiotica ingenomen?

Op ……..(dag/maand/jaar) tussen ….. en …… uur

CG5. Welke antibiotica hebt u gekregen? (Dit kunt u eventueel aflezen van de verpakking)

Naam van het middel: ..................

dosis (mg): .......................

aantal keer innemen: .......................

overige informatie: ......................

CG6. Door wie is de antibiotica voorgeschreven?

Huisarts, ...............(naam)

...............(plaats)

CG7. Waar heeft u de antibiotica opgehaald of gekocht?

Apotheek: ………..(naam)

…………(plaats)

Deelnemers uit de niet-behandelgroep welke WEL AB hebben genomen ( CG1= a / CG2=a / CG3=a) worden ingedeeld in de WEL-AB-groep (uitnodiging voor 1mnd-vragenlijst gaat hiervan uit)

Voor de deelnemers ingedeeld in de behandel groep:

BG1. Heeft u sinds de vorige vragenlijst uw huisarts bezocht om preventieve antibiotica te vragen voor uw tekenbeet?

a. Ja

b. Nee, ik heb geen antibiotica gevraagd, omdat:……..…… Ga naar: Diagnoses

BG2. Heeft u inderdaad antibiotica voorgeschreven gekregen van uw huisarts?

a. Ja

b. Nee, omdat:………. Ga naar: Diagnoses

BG3. Heeft u de antibiotica ingenomen?

a. Ja

b. Nee, vul hier de reden in:................ Ga naar: Diagnoses

BG4. Wanneer heeft u de eerste pil antibiotica ingenomen?

Op ……..(dag/maand/jaar) tussen ….. en …… uur

BG5. Welke antibiotica hebt u gekregen? (Dit kunt u eventueel aflezen van de verpakking)

Naam van het middel: ..................

dosis (mg): .......................

aantal pillen: ……………………

aantal keer innemen: .......................

overige informatie: ......................

BG6. Door wie is de antibiotica voorgeschreven?

Huisarts, ...............(naam)

...............(plaats)

BG7. Waar heeft u de antibiotica opgehaald of gekocht?

Apotheek: …………(naam)

…………(plaats)

Deelnemers uit de behandelgroep welke GEEN AB hebben genomen ( BG1= b / BG2=b / BG3=b) worden ingedeeld in de GEEN-AB-groep

#### Bijwerkingen

Dit hoofdstuk is alleen voor de WEL-AB-groep (BG3 = A |CG3 = A)

De volgende vragen gaan over mogelijke bijwerkingen door de antibiotica die u heeft ingenomen na uw tekenbeet.

BG8. Heeft u door het innemen van de antibiotica last van (mogelijke) bijwerkingen gehad? (meerdere antwoorden mogelijk)

a. Misselijkheid

b. Braken

c. Buikpijn

d. Diarree

e. Duizeligheid

f. Andere bijwerkingen namelijk: ........

g. Ik heb geen last gehad van bijwerkingen Ga naar:Diagnoses

BG9. Begindatum van het optreden van de bijwerking ………………

BG10. Hoeveel tijd zat er tussen het innemen van de antibiotica en het begin van de bijwerking?

Aantal: eenheid (minuten/uren/dagen)

BG11. Hoe is het afgelopen met deze bijwerking?

a. Hersteld, op ………… (datum)

b. Herstellende, op ………… (datum)

c. hersteld met restverschijnselen;nl:……, op ………… (datum)

d. niet-hersteld

e. anders nl,.............................................., op ………… (datum)

BG12. Is de bijwerking behandeld door een arts?

a. Ja, namelijk:………..

b. Nee

BG13. Zijn er mogelijk andere oorzaken of omstandigheden die de bijwerking(en) kunnen hebben veroorzaakt of verergerd?

a. Ja, namelijk: ……..

b. Nee

#### Diagnoses (kort)

210. Heeft u sinds de vorige vragenlijst nog nieuwe tekenbeten opgemerkt?

(is eigenlijk gelijk aan q125)

a. Nee, sindsdien heb ik geen tekenbeten opgemerkt ga naar vraag: Q213

b. Ja

211. Heeft u al deze nieuwe tekenbeten gemeld via uw account op Tekenradar.nl? (=q126)

a. Nee

b. Ja ga naar vraag: 213

212. Hoeveel tekenbeten heeft u sinds de vorige vragenlijst opgemerkt die nog niet gemeld zijn via uw account op Tekenradar.nl? (=q127)

a. 1 - 3 tekenbeten

b. 4 - 10 tekenbeten

c. 11 - 50 tekenbeten

d. Meer dan 50 tekenbeten

213. Heeft een arts bij u sinds de vorige vragenlijst een (nieuwe) erythema migrans vastgesteld?

a. Nee

b. Ja, EM

c. Ja, andere klachten door de ziekte van Lyme

deze personen worden doorgeleid naar de losse (Actieve) EM melding ->gehele EMmelding doorlopen!!!

#### Afsluiting

Afsluitings tekst!!!!!!

T = 1 maand

### Vervolgvragenlijst op T=1 maand

Voor: onderzoeksdeelnemers met startpunt = TB onafhankelijk of al een EM gemeld is

Uitnodiging gaat via een e-mail met daarin een direct link naar de vragenlijst

Hiervan bestaan 2 versies: Geen-AB-groep (CG3=b|BG3=b) Ga naar:

Wel-AB-groep (CG3=a|BG3=a) Ga naar:

#### Antibiotica (AB-groep)

Voor deelnemers die WEL antibiotica genomen hebben

Deelnemers die geen AB genomen hebben krijgen deze vragenlijst niet te zien

Hieronder worden een aantal vragen gesteld over de afgelopen 3 weken.

AB1. Heeft u sinds de vorige vragenlijst opnieuw uw huisarts bezocht voor uw tekenbeet?

a. Ja, omdat…………. (optioneel)

b. Nee Ga naar: Bijwerkingen

AB2. Heeft uw huisarts antibiotica voorgeschreven?

a. Ja, omdat………… (optioneel)

b. Nee Ga naar: Bijwerkingen

AB3. Heeft u de antibiotica ingenomen?

a. Ja

b. Nee, omdat................(optioneel) Ga naar: Bijwerkingen

AB4. Wanneer heeft u de (eerste) dosis antibiotica ingenomen?

Op ……..(dag/maand/jaar) tussen ….. en …… uur

AB5. Welke antibiotica hebt u gekregen? (Dit kunt u eventueel aflezen van de verpakking)

Naam van het middel: ..................

dosis (mg): .......................

aantal keer innemen: .......................

overige informatie: ......................

AB6. Door wie is de antibiotica voorgeschreven?

Huisarts, naam: ...............

plaats: ............

AB7. Waar heeft u de antibiotica opgehaald of gekocht?

Apotheek: ………..(naam)

…………(Plaats)

#### Antibiotica (geen-AB-groep)

Voor deelnemers die GEEN antibiotica genomen hebben

Deelnemers die wel AB genomen hebben krijgen deze vragenlijst niet te zien

AB1. Heeft u sinds de vorige vragenlijst uw huisarts bezocht voor uw tekenbeet?

a. Ja, omdat…………. (optioneel)

b. Nee Ga naar:Diagnoses

AB2. Heeft uw huisarts antibiotica voorgeschreven?

a. Ja, omdat………… (optioneel)

b. Nee Ga naar:Diagnoses

AB3. Heeft u de antibiotica ingenomen?

a. Ja

b. Nee, omdat................(optioneel) Ga naar: Diagnoses

AB4. Wanneer heeft u de (eerste) dosis antibiotica ingenomen?

Op ……..(dag/maand/jaar) tussen ….. en …… uur

AB5. Welke antibiotica hebt u gekregen? (Dit kunt u eventueel aflezen van de verpakking)

Naam van het middel: ..................

dosis (mg): .......................

aantal keer innemen: .......................

overige informatie: ......................

AB6. Door wie is de antibiotica voorgeschreven?

Huisarts, naam: ...............

plaats: ............

AB7. Waar heeft u de antibiotica opgehaald of gekocht?

Apotheek: ………..(naam)

…………(Plaats)

#### Bijwerkingen

Alleen voor deelnemers die antibiotica genomen hebben (BG3 = A |CG3 = A | AB3 = A)

Vragenlijst is identiek aan 1wk : Ga naar Bijwerkingen

Deelnemers die geen AB genomen hebben krijgen deze vragenlijst niet te zien

#### Diagnoses (kort)

Voor alle deelnemerS: Identiek aan Diagnoses(kort op T=1 wk) Ga naar: Diagnoses (kort)

#### Afsluiting ???

Er zijn 4 versies voor de afsluiting: -teek + toestemmingsverklaring is binnen

-teek is niet binnen, toestemmingsverklaring wel

-teek is binnen, toestemmingsverklaring niet

-geen teek noch toestemmingsverklaring

Hartelijk dank voor het invullen van deze vragenlijst

Indien teek + toestemmingsverklaring is binnengekomen

De door u opgestuurde teek en toestemmingsverklaring zijn door ons ontvangen. Binnen 9 maanden hoort u van ons per e-mail of de teek besmet was met de *Borrelia* bacterie die de ziekte van Lyme kan veroorzaken.

Indien alleen toestemmingsverklaring is binnengekomen

De door u opgestuurde toestemmingsverklaring is door ons ontvangen. Helaas hebben wij nog geen teek van u ontvangen. Zou u deze alsnog willen opsturen?

Q214. a. Ja

b. Nee, graag hier de reden voor aangeven:............................(optioneel)

c. Ik heb de teek al verstuurd.

Het identificatienummer van uw teek is: xxxxTRxxxx zou u dit bij de teek willen vermelden samen met uw adresgegevens. De instructies voor het opsturen kunt u eventueel hier terugvinden.

Indien alleen de teek is binnengekomen

Helaas hebben wij nog niet een ondertekende toestemmingsverklaring van u mogen ontvangen. Zou u deze zo snel mogelijk op willen sturen?

Q215. a. Ja

b. Nee, graag hier de reden voor aangeven:............................(optioneel)

c. Ik heb de toestemmingsverklaring al verstuurd.

Bent u de e-mail met de toestemmingsverklaring kwijt dan kunt u hier nogmaals een blanco formulier vinden. Dit kunt u na printen met pen invullen en ondertekenen en vervolgens sturen aan:

RIVM

Tekenradar (pb 75)

Antwoordnummer 3266

3720 VB BILTHOVEN

De door u opgestuurde teek hebben wij wel ontvangen. Indien u ook de toestemmingsverklaring nog toestuurt zullen wij binnen 9 maanden de teek testen en hoort u van ons per e-mail of de teek besmet was met de *Borrelia* bacterie die de ziekte van Lyme kan veroorzaken

Indien noch de teek noch de toestemmingsverklaring is binnengekomen

Helaas hebben wij nog geen ondertekende toestemmingsverklaring van u mogen ontvangen

Zou u deze zo snel mogelijk op willen sturen?

Q215. a. Ja

b. Nee, vul hier de reden in:............................(optioneel) vriendelijker formuleren

c. Ik heb de toestemmingsverklaring al verstuurd.

Bent u de e-mail met de toestemmingsverklaring kwijt dan kunt u hier nogmaals een blanco formulier vinden. Dit kunt u na printen met pen invullen en ondertekenen en vervolgens sturen aan:

RIVM

Tekenradar (pb 75)

Antwoordnummer 3266

3720 VB BILTHOVEN

Ook hebben wij nog geen teek van u ontvangen. Zou u deze alsnog willen opsturen?

Q214. a. Ja

b. Nee, vul hier de reden in:............................(optioneel)

c. Ik heb de teek al verstuurd.

Het identificatienummer van uw teek is: xxxxTRxxxx zou u dit bij de teek willen vermelden samen met uw adresgegevens. De instructies voor het opsturen kunt u eventueel **hier** terugvinden.

U kunt de teek ook op het daarvoor bestemde vakje van de toestemmingsverklaring plakken.

Voor iedereen:

Heeft u in de afgelopen maand een erythema migrans gekregen? Klik dan **hier** om de erythema migrans te melden en een foto te uploaden [klikbare knop naar actieve EM melding].

+Opmerkingen veld + link naar contact voor vragen (standaard form )

T = 3 maanden en verder

### Vervolgvragenlijst op T= 3,6,9,12,15,18 maanden (geen EM, geenLyme)

Voor: onderzoeksdeelnemers met startpunt = TB

Welke geen EM of lyme hebben gemeld of melden in de vervolgflow

(Q16=

Uitnodiging gaat via een e-mail met daarin een direct link naar de vragenlijst

#### 3 maanden

De volgende vragen gaan over mogelijke tekenbeten opgelopen sinds het invullen van de vorige vragenlijst 3 maanden geleden

Q125. Heeft u sinds u de vorige vragenlijst 3 maanden geleden invulde nieuwe tekenbeten opgemerkt?

a. nee, sindsdien heb ik geen tekenbeten opgemerkt >ga naar: Q128

b. ja

Q126. Heeft u al deze nieuwe tekenbeten gemeld via uw account op tekenradar.nl?

a. nee

b. ja >ga naar: Q128

Q127. Hoeveel tekenbeten heeft u sinds u de vorige vragenlijst 3 maanden geleden invulde opgemerkt die nog niet gemeld zijn via uw account op tekenradar.nl?

a. 1 - 3 tekenbeten

b. 4 - 10 tekenbeten

c. 11 - 50 tekenbeten

d. meer dan 50 tekenbeten

#### Diagnoses (lang)

De volgend vragen gaan over nieuwe manifestaties van de ziekte van Lyme die bij u zijn vastgesteld ontstaan zijn sinds het invullen van de vorige vragenlijst 3 maanden geleden. Indien u in de vorige vragenlijst,3 maanden geleden, een erythema migrans heeft gemeld en sindsdien geen nieuwe klachten heeft ondervonden van de ziekte van Lyme, kruis dan bij de eerste vraag “nee” aan. Indien u een erythema migrans of andere uiting van ‘de ziekte van Lyme’ heeft ontwikkeld na het invullen van de vorige vragenlijst, maar deze wel al heeft gemeld, kruis dan bij de eerste vraag “ja” aan. Er zullen u enkele aanvullende vragen gesteld worden, over deze nieuwe uitingen van ‘de ziekte van Lyme’.

Q128. Heeft een arts bij u de ziekte van Lyme vastgesteld sinds de vorige vragenlijst in de afgelopen 3 maanden geleden? (bijvoorbeeld een (nieuwe) erythema migrans – ook als u die al gemeld hebt in de afgelopen 3 maanden – of andere uitingsvormen van de ziekte van Lyme)?

a. nee > ga naar: einde van dit hoofdstuk

b. ja

[Als men hier ‘ja’ invult verschuift men naar de groep EM-melders]

Q129. De arts die de ziekte van Lyme vaststelde:

a. huisarts

b. bedrijfsarts

c. specialist

d. ander soort arts, namelijk:……………

Q130. Hoe is bij u de ziekte van Lyme vastgesteld? Aan de hand van:

a. alleen klinische kenmerken (symptomen/ziekteverschijnselen) > ga naar q133

b. alleen laboratoriumuitslag

c. een combinatie van klinische kenmerken en laboratoriumuitslagen

Q131. Welke laboratoriumtest is gebruikt om bij u de ziekte van Lyme vast te stellen?(meerdere antwoorden mogelijk)

a. bloedtest: ELISA en/of Western Blot (antistof bepaling in het bloed)

b. punctie of biopt van weefsel

c. PCR

d. kweek

e. andere test namelijk:……………………….

f. niet van toepassing

g. weet ik niet

Q132. Bleek uit de laboratoriumtesten dat u de ziekte van Lyme heeft/had?

a. Ja

b. nee

c. weet niet

Q133. In welk land is bij u de ziekte van Lyme vastgesteld? (meerdere antwoorden mogelijk)

a. in Nederland

b. in het buitenland, namelijk:……………..

Q134. Op welke datum is (bij benadering) de ziekte van Lyme bij u vastgesteld door een arts.

Dag maand jaar ____/______/_______

[Dag is niet noodzakelijk]

Q250 Op welke datum waren (bij benadering) de eerste gezondheidsklachten door de ziekte van Lyme (inclusief erythema migrans) begonnen?

Dag maand jaar ____/______/_______

[Dag is niet noodzakelijk]

Q135. Welke uitingsvorm(en) van de ziekte van Lyme zijn bij u vastgesteld? (meerdere antwoorden mogelijk)

a. erythema migrans (uitbreidende rode ring of vlek op de huid)

b. Borrelia-lymfocytoom (blauwrode huidverdikking vaak op oorlel door Lyme)

c. neuroborreliose (hersenvlies- en/of zenuwontsteking door Lyme)

d. Lyme-artritis (gewrichtsontsteking door Lyme)

e. Lyme-carditis (hartritmestoornissen en/of hartontsteking door Lyme)

f. acrodermatitis chronica atrophicans (ACA: huidletsel met zwelling, vaak aan onderbeen en enkels door Lyme)

g. oculaire manifestaties (ontsteking van het oog op verschillende plekken door Lyme)

h. Lyme-encefalopathie (hersenziekte met onder andere geheugen- en concentratiestoornissen door Lyme)

i. persisterende Lyme-borreliose (ná diagnose en behandeling van Lyme: aanhoudende klachten met actieve infectie door de Borrelia bacterie)

j. persisterende klachten door de ziekte van Lyme zonder aantoonbare infectie (ná diagnose en behandeling van Lyme: aanhoudende klachten zonder aantoonbare actieve infectie door de Borrelia bacterie)

k. andere diagnose, namelijk:………………..

Q251. Wat zijn de gegevens van uw huisarts?

Naam huisarts: ………………

Plaats: …………………………..

Als 135a) : de volgende vraag ook stellen:

Q136. heeft u deze erythema migrans gemeld via uw account op tekenradar.nl en een foto opgeladen?

a. nee

b.ja

[als nee dan vragen over nieuwe em hier herhalen = Uploaden foto EM / Melding EM

Mensen die een EM of andere manifestatie melden schuiven sowieso door naar de groep EM=ers]

#### Behandeling

De volgende vragen gaan over de behandeling(en) die u heeft gehad tegen de ziekte van Lyme (inclusief erythema migrans) sinds u de vorige vragenlijst heeft ingevuld 3 maanden geleden.

Q137. Heeft u antibiotica gebruikt tegen de ziekte van Lyme (inclusief erythema migrans) in de afgelopen 3 maanden?

a. nee > ga naar: einde hoofdstuk

b. weet ik niet > ga naar: einde hoofdstuk

c. ja, hoeveel kuren? …. kuren;

hoe lang was de [eerste] antibioticakuur? …..weken

[alleen als meer dan 1] tweede kuur …weken

[idem] derde kuur ….weken

[idem] vierde kuur ….weken

[vraagstelling aanpassen op basis van aantal kuren dat men invult]

Q138. Wanneer begon u met de [eerste] antibiotica behandeling voor de ziekte van Lyme (inclusief erythema migrans) in de afgelopen 3 maanden?

Q252: Datum start kuur:………….., dat was:

a. na de tekenbeet (nog voordat u ziek werd en/of voordat een erythema migrans verscheen)

b. na het verschijnen van een erythema migrans

c. na het ontstaan van andere gezondheidsklachten door de ziekte van Lyme (anders dan erythema migrans)

d. weet ik niet

[vraagstelling aanpassen op basis van het aantal kuren dat men heeft ingevuld in vraag 137.c]

Q253 Hoeveel tijd zat er tussen de eerste gezondheidsklachten (inclusief erythema migrans) en het begin van de eerste antibiotica kuur?

a. Minder dan 1 week

b. 1-2 weken

c. 2-4 weken

d. 1-2 maanden

e. 2-3 maanden

f. Langer, namelijk ….. maanden/jaren

Q139. Heeft u na de [eerste] antibioticakuur nog last gehad van gezondheidsklachten door de ziekte van Lyme (inclusief erythema migrans)?

a. ☐ nee, de klachten verdwenen

b. ☐ ja, maar de klachten waren minder ernstig

c. ☐ ja, maar de klachten namen tijdelijk af

d. ☐ ja, de ernst van de klachten is niet veranderd

e. ☐ ja, de klachten zijn erger geworden

[vraagstelling aanpassen op basis van het aantal kuren dat men heeft ingevuld in vraag 137.c]

Q140. In welk land bent u behandeld voor de ziekte van Lyme (inlclusief erythema migrans)?

a. ☐ in Nederland

b. ☐ in het buitenland, namelijk:

c. ☐ zowel in Nederland als in het buitenland, namelijk:

Q141. Door wie bent u behandeld voor de ziekte van Lyme (inclusief erythema migrans) (meerdere antwoorden mogelijk)

a. Ik ben niet behandeld

b. specialist in het ziekenhuis

c. mijn eigen huisarts

d. een huisarts gespecialiseerd in de ziekte van Lyme

e. iemand anders, namelijk:….……………..

Q142. Hebt u in de afgelopen 4 weken medicijnen gebruikt? (de anti conceptie pil niet meerekenen) Wilt u ook aanvinken of de medicijnen gegeven werden voor behandeling tegen de ziekte van Lyme, inclusief erythema migrans of om een andere reden, en of u ze tijdens een ziekenhuisopname heeft gebruikt?

a. Nee

b. Ja, namelijk ..................... medicijnen

[invul tabel met als kolommen:

• Medicijn (naam of omschrijving)

• Dosis *

• Aantal keren per dag

• Aantal dgn in de afgelopen 4 wkn

• Tegen de erythema migrans? [aankruishokje]

• Om een andere reden (klacht, ziekt of aandoening) [aankruishokje]

• Medicijnen tijdens ziekenhuisopname gebruikt [aankruishokje]]

*Als u de dosis niet weet kunt u die overslaan

[Men moet alle medicijnen in kunnen vullen, dus zo veel regels geven als nodig]

#### Klachten

Onderstaande vraag gaat over alle gezondheidsklachten die u heeft gehad in de afgelopen 3 maanden.

Q143. Welke van de onderstaande klachten heeft u gehad in de afgelopen 3 maanden? Wilt u ook aankruisen hoe lang deze klachten duurden en of de klachten tot op heden voortduren?

(meerdere antwoorden mogelijk)

Klacht Tijdsduur van uw klacht Klacht duurt voort

tot op heden

a. vermoeidheid minder dan 1 maand | 1-2 maanden | 2-3 maanden ☐

b. algehele malaise minder dan 1 maand | 1-2 maanden | 2-3 maanden ☐

c. nachtelijk zweten minder dan 1 maand | 1-2 maanden | 2-3 maanden ☐

d. verhoging minder dan 1 maand | 1-2 maanden | 2-3 maanden ☐

e. koorts (boven 38°) minder dan 1 maand | 1-2 maanden | 2-3 maanden ☐

f. grieperig gevoel minder dan 1 maand | 1-2 maanden | 2-3 maanden ☐

g. jeuk minder dan 1 maand | 1-2 maanden | 2-3 maanden ☐

h. huiduitslag minder dan 1 maand | 1-2 maanden | 2-3 maanden ☐

i. hoofdpijn minder dan 1 maand | 1-2 maanden | 2-3 maanden ☐

j. stijve nek minder dan 1 maand | 1-2 maanden | 2-3 maanden ☐

k. aangezichtspijn minder dan 1 maand | 1-2 maanden | 2-3 maanden ☐

l. aangezichtsverlamming minder dan 1 maand | 1-2 maanden | 2-3 maanden ☐

m. duizelig minder dan 1 maand | 1-2 maanden | 2-3 maanden ☐

n flauwvallen minder dan 1 maand | 1-2 maanden | 2-3 maanden ☐

o. concentratieproblemen minder dan 1 maand | 1-2 maanden | 2-3 maanden ☐

p. buikpijn minder dan 1 maand | 1-2 maanden | 2-3 maanden ☐

q. misselijkheid minder dan 1 maand | 1-2 maanden | 2-3 maanden ☐

r. darmklachten minder dan 1 maand | 1-2 maanden | 2-3 maanden ☐

s. leverklachten minder dan 1 maand | 1-2 maanden | 2-3 maanden ☐

t. oogklachten minder dan 1 maand | 1-2 maanden | 2-3 maanden ☐

u. oorklachten minder dan 1 maand | 1-2 maanden | 2-3 maanden ☐

v. spierpijn minder dan 1 maand | 1-2 maanden | 2-3 maanden ☐

w. spiertrekking minder dan 1 maand | 1-2 maanden | 2-3 maanden ☐

x. krachtverlies minder dan 1 maand | 1-2 maanden | 2-3 maanden ☐

y. rugpijn minder dan 1 maand | 1-2 maanden | 2-3 maanden ☐

z. pijnlijke gewrichten minder dan 1 maand | 1-2 maanden | 2-3 maanden ☐

aa. gewrichtsontsteking minder dan 1 maand | 1-2 maanden | 2-3 maanden ☐

bb. tinteling of pijn in armen of benen minder dan 1 maand | 1-2 maanden | 2-3 maanden ☐

cc. zenuwpijn minder dan 1 maand | 1-2 maanden | 2-3 maanden ☐

dd. hart- of vaat- klachten minder dan 1 maand | 1-2 maanden | 2-3 maanden ☐

ee. somberheid minder dan 1 maand | 1-2 maanden | 2-3 maanden ☐

ff. stemmingswisselingen minder dan 1 maand | 1-2 maanden | 2-3 maanden ☐

gg. anders namelijk: ……. minder dan 1 maand | 1-2 maanden | 2-3 maanden ☐

hh (=Q143_38). geen van bovenstaande klachten

[duur van de klachten graag aangeven via pull-down menu]

#### Algemene gezondheid

Alleen op t=12 (moet jaarlijks ingevuld worden)

Zie: Algemene gezondheid (TB-E1)

#### Afsluiting 4

Op t= 3,6,9,12,15

Hartelijk dank voor het invullen van de vragen.

Op het volgende meetmoment ontvangt u de e-mail met de link naar de volgende vragenlijst.

Mocht u in de tussentijd een tekenbeet oplopen dan kunt u dit melden op de website, en de teek opsturen. Deze tekenbeet zal vervolgens ook aan uw account gekoppeld worden. U kunt dit ook doorgeven op het moment van de volgende vragenlijst, echter voor het testen van de teek is het belangrijk dat deze zo snel mogelijk opgestuurd wordt.

Opmerkingen

#### Afsluiting 6

Op t=18

Hartelijk dank voor het invullen van de vragen.

Dit was de laatste vragenlijst voor het onderzoek. Mocht u over 3 maanden opnieuw een tekenbeet oplopen, dan kunt u nogmaals meedoen aan het onderzoek.

De resultaten van tekenradar worden regelmatig op de website en in een nieuwsbrief getoond.

Meld u hier aan voor de nieuwsbrief.

Opmerkingen

### Vervolgvragenlijst op T= 3,6,9,12,15,18 maanden (EM melder, geen Lyme)

Voor: onderzoeksdeelnemers met startpunt = TB + EM gemeld (Q16 = )

Startpunt = EM

Welke geen lyme hebben gemeld of melden in de vervolgflow (Q135 ≠ b t/m k)

Of geen nieuwe manifestatie melden op het huidige meetmoment (Q128=a)

Uitnodiging gaat via een e-mail met daarin een direct link naar de vragenlijst

#### 3 maanden

Zie: 3 maanden

#### Diagnoses (lang)

Zie: Diagnoses (lang)

Q128=a

#### Behandeling

Zie: Behandeling

#### Klachten

Zie: Klachten

#### Algemene gezondheid

Alleen op t=12 (moet jaarlijks ingevuld worden)

Zie: Algemene gezondheid (TB-E1)

#### Algemeen welbevinden 2 (rand 36)

Zie: Algemeen welbevinden (lange versie) EM-G2

#### Zorgconsumptie

Zie: Zorgconsumptie (EM-G5)

#### Ziekteverzuim

Zie: Ziekteverzuim (EM-G6)

#### Afsluiting 4

Op t= 3,6,9,12,15

Hartelijk dank voor het invullen van de vragen.

Op het volgende meetmoment ontvangt u de e-mail met de link naar de volgende vragenlijst.

Mocht u in de tussentijd een tekenbeet oplopen dan kunt u dit melden op de website, en de teek opsturen. Deze tekenbeet zal vervolgens ook aan uw account gekoppeld worden. U kunt dit ook doorgeven op het moment van de volgende vragenlijst, echter voor het testen van de teek is het belangrijk dat deze zo snel mogelijk opgestuurd wordt.

Opmerkingen

#### Afsluiting 6

Op t=18

Hartelijk dank voor het invullen van de vragen.

Dit was de laatste vragenlijst voor het onderzoek. Mocht u over 3 maanden opnieuw een tekenbeet oplopen, dan kunt u nogmaals meedoen aan het onderzoek.

De resultaten van tekenradar worden regelmatig op de website en in een nieuwsbrief getoond.

Meld u hier aan voor de nieuwsbrief.

Opmerkingen

### Vervolgvragenlijst op T= 3,6,9,12,15,18 maanden (nieuwe EM|Lyme of ooit Lyme)

Voor: onderzoeksdeelnemers met startpunt = TB of EM

Welke nu een nieuwe EM of Lyme melden(Q128 = B in deze vragenlijst)

Of ooit een Lyme hebben gemeld (Q136 = b t/m k in deze vragenlijst of ooit eerder)

Uitnodiging gaat via een e-mail met daarin een direct link naar de vragenlijst

#### 3 maanden

Zie: 3 maanden

#### Diagnoses (lang)

Zie: Diagnoses (lang)

#### Behandeling

Zie: Behandeling

#### Klachten

Zie: Klachten

#### Algemene gezondheid

Alleen op t=12 (moet jaarlijks ingevuld worden)

Zie: Algemene gezondheid (TB-E1)

#### Algemeen welbevinden 2 (rand 36)

Zie: Algemeen welbevinden (lange versie) EM-G2

#### Klachten perceptie

Zie: Klachtenperceptie (EM-G4)

#### Verwachtingen klachtenbeloop

Zie: Verwachtingen klachtenbeloop (EM-G3)

#### Zorgconsumptie

Zie: Zorgconsumptie (EM-G5)

#### Ziekteverzuim

Zie: Ziekteverzuim (EM-G6)

#### Afsluiting 4

Op t= 3,6,9,12,15

Hartelijk dank voor het invullen van de vragen.

Op het volgende meetmoment ontvangt u de e-mail met de link naar de volgende vragenlijst.

Opmerkingen

***Afsluiting 6***

Op t=18

Dit was de laatste vragenlijst van het onderzoek. Hartelijk dank voor uw medewerking de afgelopen tijd. Mocht u over meer dan 3 maanden opnieuw een tekenbeet oplopen, dan kunt u nogmaals meedoen aan het onderzoek.

De resultaten van tekenradar worden op de website en in een nieuwsbrief gerapporteerd.

Meld u **hier** aan voor de nieuwsbrief.

Opmerkingen

## E-mail teksten

### Nieuwe account activeren

Titel: Account activeren

Beste voornaam prefix ahternaam,

Dank voor je inschrijving.

Volg deze link om je inschrijving te bevestigen: www.tekenradar.nl/mijn-tekenradar?action=RegisterConfirm&id=7170&t=7dfa671adece65e4ca92d26d86691c142d548a25

Vragen of opmerkingen? Mail naar info@tekenradar.nl

### Bevestiging anonieme melding

Titel = Bedankt voor uw melding van een tekenbeet op Tekenradar.nl

Geachte heer/mevrouw,

Hartelijk dank voor het melden van uw tekenbeet op Tekenradar.nl.

Klik hier om uw tekenbeet melding te bevestigen.

Houdt u de plek van de tekenbeet de komende drie maanden goed in de gaten. Mocht hier een rode ring (erythema migrans) verschijnen of mocht u andere klachten krijgen die kunnen duiden op de ziekte van Lyme, dan adviseren wij u contact op te nemen met uw huisarts.

Indien Q????= a

Over drie maanden sturen wij u per e-mail een herinnering om u te vragen of u een erythema migrans heeft ontwikkeld.

Vriendelijke groeten,

Tekenradar.nl

Tekenradar.nl is een samenwerking van Wageningen University, RIVM en De Natuurkalender.

Klik hier om een nieuwe tekenbeet te melden.

Klik hier om een erythema migrans te melden.

### Bevestiging melding tekenbeet (+ deelname in behandelgroep)

Titel = Dank voor uw deelname aan de Tekenradar studie

Geachte heer/mevrouw voornaam prefix achternaam,

Dank voor uw deelname aan de studie. U bent ingedeeld in de **behandelgroep**.

Daarom vragen wij u zo snel mogelijk een afspraak te maken met uw huisarts voor preventieve antibiotica. Als attachment bij deze email ontvangt u informatie voor uw huisarts. Deze informatie kunt u uitprinten en meenemen bij de afspraak. Hierin staat namelijk welke richtlijnen de huisarts moet volgen voor behandeling en welk medicijn hij moet voorschrijven.

Ook vindt u als attachment in deze email nogmaals de informatie voor deelnemers en de toestemmingsverklaring. Wij vragen u de toestemmingsverklaring direct te printen en te ondertekenen, mocht u dat nog niet gedaan hebben. Stuur de toestemmingsverklaring liefst samen met de teek vandaag nog op naar onderstaand antwoordnummer (geen postzegel nodig).

RIVM

Tekenradar (pb 75)

Antwoordnummer 3266

3720 VB BILTHOVEN

Houdt u de plek van de tekenbeet de komende drie maanden goed in de gaten. Mocht hier een rode ring (erythema migrans) verschijnen of mocht u andere klachten krijgen die kunnen duiden op de ziekte van Lyme, dan adviseren wij u opnieuw contact op te nemen met uw huisarts.

Over 1 week sturen wij u per e-mail een uitnodiging voor het invullen van een vervolgvragenlijst.

Vriendelijke groeten,

Tekenradar.nl

### Bevestiging melding EM

### Bevestinging account Tekenradar.nl

### Uitnodiging vervolgvragenlijst

### Reminder voltooien vragenlijst

Indien iemand de vragenlijst niet volledig voltooid, maar deze halverwege dichtklikt krijgt hij 2 dagen later een reminder met de vraag om de vragenlijst alsnog te voltooien.

Geachte Test re minder,

Helaas is uw melding op Tekenradar van 2 dagen geleden nog niet geheel voltooid, omdat u de vragenlijst nog niet volledig heeft afgerond. Via deze e-mail willen wij u uitnodigen om dat alsnog te doen.

Klik hier om de vragenlijst te hervatten en af te ronden.

Met vriendelijke groeten,

Tekenradar.nl

Tekenradar.nl is een samenwerking van Wageningen University, RIVM en De Natuurkalender

### Reminder vervolgvragenlijst
